# Hijacking Endocytosis and Autophagy in Extracellular Vesicle Communication: Where the Inside Meets the Outside

**DOI:** 10.3389/fcell.2020.595515

**Published:** 2021-01-07

**Authors:** Giona Pedrioli, Paolo Paganetti

**Affiliations:** ^1^Neurodegeneration Research Group, Laboratory for Biomedical Neurosciences, Neurocenter of Southern Switzerland, Ente Ospedaliero Cantonale, Torricella-Taverne, Switzerland; ^2^Member of the International Ph.D. Program of the Biozentrum, University of Basel, Basel, Switzerland; ^3^Faculty of Biomedical Sciences, Università della Svizzera Italiana, Lugano, Switzerland

**Keywords:** aggregation, autophagy, cargo, cell-to-cell communication, endocytosis, extracellular vesicles, lysosome, neurodegeneration

## Abstract

Extracellular vesicles, phospholipid bilayer-membrane vesicles of cellular origin, are emerging as nanocarriers of biological information between cells. Extracellular vesicles transport virtually all biologically active macromolecules (e.g., nucleotides, lipids, and proteins), thus eliciting phenotypic changes in recipient cells. However, we only partially understand the cellular mechanisms driving the encounter of a soluble ligand transported in the lumen of extracellular vesicles with its cytosolic receptor: a step required to evoke a biologically relevant response. In this context, we review herein current evidence supporting the role of two well-described cellular transport pathways: the endocytic pathway as the main entry route for extracellular vesicles and the autophagic pathway driving lysosomal degradation of cytosolic proteins. The interplay between these pathways may result in the target engagement between an extracellular vesicle cargo protein and its cytosolic target within the acidic compartments of the cell. This mechanism of cell-to-cell communication may well own possible implications in the pathogenesis of neurodegenerative disorders.

## Introduction

In multicellular organisms, cell-to-cell communication is a cardinal process that coordinates and synchronizes cellular activities, ensuring the correct function of tissues, organs, and ultimately the whole system. A broad variety of mechanisms has evolved to accomplish the transmission of information between neighboring or distant cells. Among these, lipid bilayer-membrane nanovesicles released from cells act as vectors for short- and long-distance transport of biological messages. First described in the early 1980s as secreted exosomes derived from endosomes (Harding et al., 1983; Pan and Johnstone, 1983; Pan, 1985), their function was first proposed as an alternative degradative path through which cells expel dispensable intracellular molecules. The subsequent advances in isolation and characterization procedures depicted a more complex and heterogeneous population of secreted nanovesicles, for which the generic term “extracellular vesicles” (EVs) was coined (György et al., 2011). The first concept on their function was challenged by the observation that EVs secreted from B cells induce T-cell proliferation by activating major histocompatibility complex II (MHC-II) receptors carried on their surface (Raposo et al., 1996). More recently, a strong support for a relevant role of macromolecules transported by EVs in cell-to-cell communication is reflected by the demonstration that nucleic acids synthetized and encapsulated in EVs by the donor cells are active in recipient cells (Valadi et al., 2007). It is now established that EVs are required for multiple cellular processes in health and disease by contributing to immunomodulation, inflammation, cancer, and neurodegeneration (Mathieu et al., 2019). EVs even find applications as nanocarriers for therapeutic agents (Luan et al., 2017; Yang et al., 2018).

At least three main criteria can be defined for EVs to be accounted as functional vectors in cell-to-cell communication: secretion by the donor cell after selective macromolecule encapsulation, transport of the cargo to the target cell, and release of the transported messenger in the recipient cell for interaction with its effector. To date, the efforts in the field were primarily directed to understand the biogenesis and the mechanisms for the packaging of macromolecules in EVs (Raposo and Stoorvogel, 2013; Colombo et al., 2014; van Niel et al., 2018; Mathieu et al., 2019). EVs comprise small EVs (with a diameter of 30–150 nm) or large EVs (>150 nm) that mostly depend on their origin from endosomes or the cell surface, respectively (Colombo et al., 2014). Exosomes, a class of small EVs, are born as intraluminal vesicles (ILVs) by inward membrane budding during the maturation of multivesicular bodies (MVBs), a process that is regulated by the endosomal sorting complex required for transport (ESCRT) (Colombo et al., 2014; Cocucci and Meldolesi, 2015; Mathieu et al., 2019). The ESCRT-0 and ESCRT-I subcomplexes sort cargo molecules in membrane microdomains, and the ESCRT-II and ESCRT-III subcomplexes drive membrane budding and fission. ILVs are also generated by ESCRT-independent mechanisms. This can occur by the hydrolysis of sphingomyelin to ceramide and the creation of membrane microdomains; further metabolism of ceramide to sphingosine 1-phosphate activates its receptor that sorts the cargo of ILVs (Colombo et al., 2014). In addition, members of the tetraspanin family, which are efficiently sorted to the endosomal pathway, appears involved in the sorting of EV cargo (van Niel et al., 2011). The fusion of MVBs at the cell surface results in exosome secretion. Large EVs, in particular microvesicles, predominantly originates from the outward budding of the plasma membrane (van Niel et al., 2011). Cargo packaging entails that macromolecules are first targeted to the respective production site. Then, with a process requiring molecular clustering and budding, they end up in EVs following membrane fission. The proteins involved in the biogenesis of EVs eventually become themselves cargo molecules and can be utilized as markers characterizing the origin of EVs (Colombo et al., 2014; Mathieu et al., 2019). On the other hand, although the delivery of cargo macromolecules to recipient cells is a critical step required for absolving the biological activities associated to EVs, the mechanisms involved in this process remain largely elusive. Macromolecules transported on the exterior of EV may directly interact with surface membrane receptors, as it may be the case for MHC-II receptors. For a luminal EV cargo, it is plausible to assume that the cytosol is the main site for intracellular target engagement. However, current evidence suggests that this is a possible but rather rare event (Ridder et al., 2014; Zomer et al., 2015; Sterzenbach et al., 2017). Inadequate detection sensitivity is a plausible technical limitation. In fact, circumstantial evidence indicates that a measurable biological effect is counterbalanced by the difficulty to detect a cargo molecule in the cytosol of the recipient cell (Zomer et al., 2015; Steenbeek et al., 2018; Pedrioli et al., 2020). Lack of sensitivity may result because recipient cells represent only a minor subpopulation able to decipher an EV message. In such a case, EVs specifically targeting a specialized cell pool present in an organ may reduce EV cargo release to a rare event that still holds a significant biological relevance (Ridder et al., 2014; Zomer et al., 2015). At the same time, a sporadic but continuous transport over years of pathological protein forms between cells may well contribute to a slow progression and propagation of disease as in the case of neurodegenerative disorders. That being said, an infrequent cytosolic EV cargo release does not exhaustively explain the large body of evidence, suggesting a cardinal role of EVs in cell-to-cell exchange of macromolecules. Nevertheless, given the broad heterogeneity of luminal cargos, there is the need to assess whether alternative intracellular locations may account for the release and the target engagement of biologically active EV cargo macromolecules.

## Looking for the Target Cell

The rising interest around EVs in recent years is linked to the increasing evidence of phenotypic changes in recipient cells apt to translate a message transported by these vesicles (Valadi et al., 2007; Ridder et al., 2014; Zomer et al., 2015; van Niel et al., 2018; Mathieu et al., 2019; Wang et al., 2019; O’Brien et al., 2020). The molecular process exploited by EVs to target recipient cells remains a matter of debate, possibly because different mechanisms may coexist. Human carcinoma cells were shown to non-selectively respond to EVs originating from different cell types (Horibe et al., 2018). The composition and modifications of external components may affect the overall charge of the EV surface, thus reducing the natural electrostatic repulsion of membranes (Williams et al., 2019). This process may become more relevant once EVs are internalized into the acid environment of endocytic organelles (Winchester, 2005). Besides a randomly determined event, a combination of EV and cell origin, EV subtype, and cell type and state may confer specificity to the recognition of EVs by the recipient cell, a mechanism defined as tropism of EVs (Kooijmans et al., 2016). EVs derived from B cells in mantle cell lymphoma (MCL) are readily and preferentially taken up by other MCL cells (Hazan-Halevy et al., 2015). In the nervous system, EVs secreted from oligodendrocytes have a specific tropism for microglia cells (Fitzner et al., 2011). In both cases, preferential EV internalization may occur because of active intake mechanisms characterizing these cell types. EV docking at the plasma membrane may be facilitated by cell membrane adhesion receptors recognizing macromolecules exposed on the surface of EVs. Tetraspanins, in particular CD9 and CD81, which are highly enriched in the lipid membranes of EVs, appear as possible candidates (Morelli et al., 2004). Additional proteins exposed on the EV surface participate in ligand-binding mechanisms. On dendritic cell–derived EVs, the beta-2 integrin family of proteins (CD18/CD11 a, b), the intercellular adhesion molecule-1 and -2 (ICAM-1/-2), and the serum milk fat globule-EGF factor 8 facilitate the interaction with recipient cells (Théry et al., 1999, 2001; Nolte-‘T Hoen et al., 2009; Genschmer et al., 2019). Glycans as well may contribute to the EV and cell recognition process (Williams et al., 2018, 2019; Dusoswa et al., 2019). Glioblastoma-derived EVs are decorated with glycans recognized by sialic acid–binding immunoglobulin-like lectin receptors, an essential and specific step for their capture by dendritic cells (Dusoswa et al., 2019). To add complexity to the system, the heterogeneous size and composition of EVs may influence their recognition and uptake by recipient cells.

As all cell types secrete EVs, the extracellular milieu is rich in a large variety of EVs. A productive message possibly covering a distant radius of action requires that the target cell developed a precise instrument of docking and internalization of freely circulating EVs. The elucidation of the fate of EVs once docked on the cell surface is of critical importance in the context of disease. Understanding the molecular and cellular mechanisms involved in a pathogenic cell-to-cell communication mediated by EVs may offer new approaches for the development of specific treatments.

## “Eating and Drinking” EVs

The variability in EV uptake routes may depend on the combination of multiple factors contributed by macromolecules present on the surface of both EVs and recipient cells (Mulcahy et al., 2014). Most experimental evidence suggests that endocytosis is the major uptake path (Koumangoye et al., 2011; Nanbo et al., 2013; Mulcahy et al., 2014; Heusermann et al., 2016; Nakase et al., 2016; Durak-Kozica et al., 2018; Yao et al., 2018). EVs are internalized by dendritic cells and fuse with membranes of the endocytic pathways releasing their content into the cytosol (Montecalvo et al., 2012). However, once taken up by recipient cells, EVs can also be either recycled and released in the extracellular space or targeted to lysosomes for degradation. For instance, upon internalization by interconnected neurons, fusion events between exogenous and endogenous EVs were found to potentially increase the radius of action of EVs and the consequent pathogenicity in the context of Alzheimer disease (AD) (Polanco et al., 2018). In contrast, microglia take up oligodendrocyte-derived EVs through a macropinocytotic mechanism on their route to lysosomes for degradation (Fitzner et al., 2011), consistent with their role in cleaning the extracellular space from cell debris.

**FIGURE 1:**
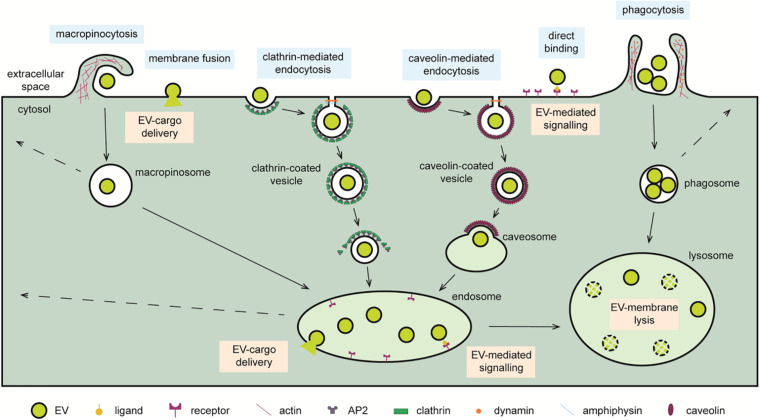
Cellular pathways exploited for the delivery of EV cargo. EVs reaching recipient cells can interact with cell surface receptor or fuse with the limiting membrane and deliver the soluble cargo directly to the cytosol. Alternatively, EVs are internalized through macropinocytosis, micropinocytic processes such as clathrin-mediated endocytosis, and caveolin-mediated endocytosis or phagocytosis. Internalized EVs transit through endosomal compartments when directed to lysosomes. Within endo-lysosomal organelles, ligands present on the EV surface can induce an intracellular signaling cascade through a ligand–receptor mechanism. Moreover, cytosolic delivery of EV cargo may occur by fusion with the membrane of these organelles. The action of acidic hydrolases may liberate the EV cargo for degradation, interaction with other endo-lysosomal components, or recycling to the extracellular milieu by back fusion with the cell membrane. Symbols used are specified in the legend on the bottom of the scheme.

The term “endocytosis” was coined by Christian de Duve in the 1960s to describe a cellular process in which the invagination of the limiting plasma membrane leads to the intracellular formation of vesicles encapsulating extracellular material (Fürthauer and Smythe, 2014). Various functions are now assigned to endocytosis, a key homeostatic mechanism that regulates major cellular processes such as provision of educts for biochemical synthesis of macromolecules, receptor down-regulation, intracellular signaling, antigen presentation (Miaczynska et al., 2004; Miaczynska and Stenmark, 2008; Ellinger and Pietschmann, 2016), or as the main route for EV internalization (Koumangoye et al., 2011; Nanbo et al., 2013; Mulcahy et al., 2014; Heusermann et al., 2016; Nakase et al., 2016; Durak-Kozica et al., 2018; Yao et al., 2018). Indeed, EV uptake through this route is rapid, with EVs identified inside cells within few minutes after their application to the culture medium (Feng et al., 2010). EV uptake requires an active process as shown by its absence in cells kept at 4°C or fixed with paraformaldehyde (Fitzner et al., 2011; Pan et al., 2012) and is therefore modulated by the same mechanisms regulating endocytosis (Joseph and Liu, 2020). Alternatively, it may occur by bulk flow, similarly, to the fluid phase-uptake marker dextran (Tian et al., 2014a; Nakase et al., 2016). Once internalized, EVs locate with various markers of the endocytic pathway. For instance, EVs derived from Epstein–Barr virus–infected B cells and tracked through a fluorescent lipophilic dye localize, at increasing time points, with RAB5, RAB7, and CD63-positive endocytic organelles of recipient epithelial cells (Nanbo et al., 2013). EVs are observed to enter human primary fibroblasts via filopodia and travel along the endocytic pathway and end their route, after scanning the endoplasmic reticulum, in lysosomes (Heusermann et al., 2016). Consistent with this, EVs appear to exploit the endocytic pathway to travel from the periphery, rich in early endosomes, toward the perinuclear area, rich in late endosomes and lysosomes, or they are sorted and recycled by secretion at the plasma membrane (Mercer et al., 2010; Durak-Kozica et al., 2018), as described for protein receptors (Gonda et al., 2019). Indeed, EVs (and viral particles) are found within lysosomes of recipient cells as soon as 1 h after application (Koumangoye et al., 2011; Zhou et al., 2011; Yao et al., 2018). This close proximity with the nucleus is hijacked for the delivery of viral genomic material to “the control center of the cell” and may hint to a possible shared delivery mechanism also used by EVs (Mercer et al., 2010). Endocytosis is a broad term that includes a range of internalization pathways that include cell eating (“phagocytosis”) and cell drinking (“pinocytosis”) processes, both involved in EV internalization (Feng et al., 2010; Tian et al., 2014a; Ellinger and Pietschmann, 2016; Holder et al., 2016; Chiba et al., 2018; Horibe et al., 2018; Ogese et al., 2019; Verweij et al., 2019; Figure 1).

### Phagocytosis

Phagocytosis is the main internalization path shared by professional phagocytic cells, which embrace neutrophils, macrophages, and dendritic cells (Savina and Amigorena, 2007; Richards and Endres, 2014). Phagocytosis is tightly regulated, and it requires ligand–receptor–mediated recognition followed by the active ingestion of large extracellular particles (>0.5 μm) into intracellular organelles called phagosomes. These latter are then directed to fuse with lysosomes to generate phagolysosome (Stuart and Ezekowitz, 2005; Kuhn et al., 2014; Kettler et al., 2016; Rosales and Uribe-Querol, 2017). Given the nature of this process, it is understood that phagocytes preferentially internalize large EVs, such as apoptotic bodies and ectosomes. Of particular interest is the highly selective uptake of apoptotic bodies by dendritic cells, a route that is mediated through the receptors for phosphatidylserine (PS), which is enriched on the surface of these large cell debris (Rubartelli et al., 1997; Hoffmann et al., 2001; Gasser et al., 2003). Nevertheless, also particles less than 100 nm in size are observed to be taken up by phagocytes (Kuhn et al., 2014; Kettler et al., 2016). This is supported by the observation that dendritic cells internalize exosomes through a process that is inhibited by latrunculin-A (Ogese et al., 2019), a potent inhibitor of phagocytosis (de Oliveira and Mantovani, 1988) acting by depolarizing actin filaments (Fujiwara and Zweifel, 2018). Likewise, exosomes derived from leukemia cell lines are taken up by a process sensitive to genetic inhibition of a key regulator of phagocytosis (DNM2), and once internalized, they localize with phagolysosome markers (Feng et al., 2010). Despite sufficient evidence for phagocytosis as an endocytic process involved in EV uptake, the exact mechanisms of small-size EVs phagocytosis, e.g., the receptor involved, remain to be elucidated. Moreover, given the nature of phagocytic cells, internalized EVs end up in organelles rich in digestive enzymes (Feng et al., 2010), thus representing a mean for their elimination, rather than a process involved in cell-to-cell communication.

### Pinocytosis

Pinocytosis describes a process in which inward budding of the plasma membrane serves to internalize small amounts of extracellular fluid and dissolved particles eventually forming intracellular pinocytic vesicles. Pinocytosis is subdivided in micropinocytosis (<0.1 μm) and macropinocytosis (∼0.2–5.0 μm) according to the size (and fate) of pinocytic vesicles and the molecular mechanism involved (Steinman, 1983; Kruth et al., 2005).

#### Micropinocytosis

Most receptor-bound ligands are internalized via micropinocytosis providing an efficient means for uptake of specific macromolecules (Sallusto, 1995; Banchereau and Steinman, 1998; Swanson, 2008; Mercer and Helenius, 2009; Bloomfield and Kay, 2016). The internalization of transferrin upon binding to the transferrin receptor is the best studied example of receptor-mediated endocytosis (Sullivan et al., 1976; Harding et al., 1983). Coupling transferrin with a fluorescent tag, a routine in monitoring micropinocytosis, showed different localization of EVs with transferrin, depending on incubation time and recipient cell type (Tian et al., 2014a; Yao et al., 2018). Micropinocytosis forms clathrin-, caveolin-, or non–clathrin/non–caveolin–coated plasma membrane pits (Steinman, 1983; Kruth et al., 2005; Kaksonen and Roux, 2018). Then, invaginated membranes pinch off to create organelles that mature and fuse with endosomes. This delivers their contents for recycling after fusion with the plasma membrane or for further transport to lysosomes (Mellman, 1996; Kirchhausen, 2000; Kaksonen and Roux, 2018).

EV size, EV origin, and recipient cell type may all play a role in deciding whether micropinocytosis entails the use of clathrin or caveolin or neither. EVs localize with markers of clathrin-mediated endocytosis (CLME) by a process sensitive to inhibitors of key effectors of this pathway. The compound pitstop 2 is a potent, but not selective, inhibitor of clathrin-dependent endocytosis that binds to the amino terminus of clathrin and blocks its association with amphiphysin (von Kleist et al., 2011; Dutta et al., 2012; Willox et al., 2014). CLME inhibition by pitstop 2 decreases EV uptake in human trophoblast cells (Holder et al., 2016) and colorectal carcinoma cells (Horibe et al., 2018). The cationic amphiphilic drug chlorpromazine inhibits the formation of clathrin-coated pits at the plasma membrane (Wang et al., 1993) and reduces the uptake of EVs in several *in vitro* cell culture models (Feng et al., 2010; Escrevente et al., 2011; Tian et al., 2014a; Yao et al., 2018). EV internalization is also decreased in the presence of pyrimidyn-7 and dynasore (Macia et al., 2006; McGeachie et al., 2013; Chiba et al., 2018; Verweij et al., 2019), two non-selective inhibitors of the GTPase activity of dynamin, a large GTPase involved in late stages of clathrin-coated pits formation (Sever et al., 2000; Hill et al., 2001). The genetic shRNA knockdown of clathrin adaptor protein 2 (AP2) and that of dynamin inhibit the assembly of clathrin-coated pits and result in an appreciable reduction of EV uptake (Tian et al., 2014a). While CLME is the best documented micropinocytic process for EV uptake, the involvement of caveolin-mediated endocytosis (CAME) becomes increasingly evident. Because of the small size of the caveolae, it is plausible to assume that CAME tends to internalize EVs with a small 60–80 nm diameter (Wang et al., 2009; Parton and Collins, 2016). The endogenous expression of caveolin-1, the main structural protein of caveolae, fluctuates between cancer cell lines and correlates with the degree of EV uptake (Horibe et al., 2018). In support to this, specific shRNA knockdown of caveolin-1 impairs EV internalization (Nanbo et al., 2013). Sterol-binding compounds that disrupt lipid rafts and caveolae structures, such as filipin, genistein, and nystatin, inhibit CAME-mediated EV uptake in various cell types (Svensson et al., 2013; Tian et al., 2014a; Lin et al., 2018; Yao et al., 2018; Harischandra et al., 2019). As dynamin also participates to the formation of caveolae at the plasma membrane (Oh et al., 1998), studies assessing the role of this protein through the use of genetic and pharmacological inhibitors do not discern the possible involvement of CLMA and CAME.

Taken together, the evidence that micropinocytosis is implicated in the internalization of small-size EVs is well documented. However, some of the molecular mechanisms involved in this process need more investigation, in particular the selective tropisms toward subclasses of small-size EVs.

#### Macropinocytosis

Macropinocytosis, on the other hand, overcomes the size limitation in EV internalization of micropinocytic processes. Macropinocytosis is characterized by the formation of actin-rich plasma membrane extensions, named ruffles (Swanson and Watts, 1995; Kerr and Teasdale, 2009). These pockets-like membrane structures fuse back with the plasma membrane and pinch off to form non-coated organelles, referred to as macropinosomes, which encapsulate a large volume of extracellular material (Kerr and Teasdale, 2009). The relatively large size of macropinosomes allows the uptake of a greater load of EVs and a broader range of EV sizes when compared to micropinosomes. As for most endocytic organelles, macropinosomes mature, shrink, and move toward the center of the cell where they eventually fuse with lysosomes. Although rarely, these organelles can recycle back to the plasma membrane and release their content in the extracellular space (Swanson and Watts, 1995). Macropinocytosis is an efficient, although non-selective, mechanism for internalizing EVs (Swanson and Watts, 1995). To demonstrate the participation of this pathway in the EV uptake, various inhibitors targeting the machinery generating macropinosomes were employed (Swanson and Watts, 1995; Swanson, 2008; Kerr and Teasdale, 2009; Lim and Gleeson, 2011). Macropinocytosis is dependent on the Na^+^/H^+^ exchanger (NHE) activity (Swanson and Watts, 1995; Koivusalo et al., 2010). EIPA (5-(*N*-ethyl-*N*-isopropyl)amirolide) is an inhibitor of NHE that impairs micropinocytosis and EV uptake (Tian et al., 2014a; Costa Verdera et al., 2017). Other non-selective compounds are applied to study macropinocytosis. Wortmannin and LY294002 (Feng et al., 2010; Tian et al., 2014a; Costa Verdera et al., 2017), potent inhibitors of phosphoinositide 3-kinases, impair intracellular membrane traffic and endocytosis (Araki et al., 1996). Latrunculin-A and cytochalasin-D destabilize actin filaments (Lamaze et al., 1997; Yarmola et al., 2000; Veltman et al., 2016) and inhibit ruffles formation (Montecalvo et al., 2012; Svensson et al., 2013; Emam et al., 2018). As macropinocytosis shares with phagocytosis similar molecular mechanisms, the use of inhibitors is not sufficient to infer on the specific involvement of either processes. Colocalization of EVs with fluorescently tagged dextran, a fluid-phase marker of endocytosis, is consistent with this view (Kerr and Teasdale, 2009; Fitzner et al., 2011; Tian et al., 2014a; Costa Verdera et al., 2017; Canton, 2018). Moreover, macropinocytosis does not specifically target molecules in the extracellular environment, indicating that EV uptake may be dictated just by their proximity to the cell membrane. Nevertheless, macropinocytosis is regulated by specific mechanisms (Bryant et al., 2007; Ha et al., 2016; Colin et al., 2019); stimulation, e.g., of the epidermal growth factor receptor, enhances EV uptake (Nakase et al., 2015; Colin et al., 2019). Notably, also EVs appear to induce macropinocytosis (Costa Verdera et al., 2017). Similar to phagocytosis, macropinocytosis channels EVs to lysosomes (Fitzner et al., 2011; Yao et al., 2018), possibly indicating a clearance mechanism, rather than a route for transcellular signaling.

## Delivering an EV Cargo: Membrane Fusion

Cells exploit EVs to communicate biological information over short and long distances, implying that the message transported by EVs must engage with its natural target. This may occur at the cell surface excluding an internalization process (Shelke et al., 2019), e.g., when B-lymphocyte EVs activate T-cell receptors on the surface of lymphocytes (Raposo et al., 1996). In contrast, for a cargo such as RNAs transported in the EV lumen, the target enabling a biological response is likely to be located in the nuclear-cytosolic compartment where the machineries for, e.g., mRNA translation or for microRNA regulation are expected (Valadi et al., 2007; Zomer et al., 2015). However, there is a lack of knowledge on the cell biology and biochemistry allowing the EV cargo to bypass the robust barriers imposed by the phospholipid bilayers, limiting both EVs and recipient cells. Not surprisingly, the direct fusion of EVs with the membranes of recipient cells is proposed as a relatively simple process for cell entry (Parolini et al., 2009; Zomer et al., 2015; Prada and Meldolesi, 2016; Van Dongen et al., 2016). Given the large body of evidence for an EV uptake by endocytosis before cargo delivery, it is conceivable that endocytic organelles may act as the location where EVs fuse with cell membrane, in a process known as “endosomal escape.” In this sense, recent studies offer a reasonable mechanism of membrane fusion facilitated by the acidic environment and by the degradative activity of lysosomal hydrolases (Parolini et al., 2009; Montecalvo et al., 2012; Joshi et al., 2020). This is reminiscent of the process used by several virus to deliver genomic material to the host cell (Burkard et al., 2014). Indeed, low pH and acidic hydrolases may induce conformational changes in viral fusion proteins that facilitate the merge with the cell membranes (Burkard et al., 2014; Staring et al., 2018). This is the case for the rabies virus glycoprotein (RVG), which binds to the nicotinic acetylcholine receptor expressed by cells of the nervous system (Lentz et al., 1982; Gaudin et al., 1993; Lafon, 2005).

Interestingly, liposomes and other types of synthetic EVs are developed as vectors for the delivery of membrane-impermeable drugs, whereby multiple solutions were designed to improve endosomal escape. The engineering of EVs bearing a short peptide derived from RVG efficiently delivers functional BACE1 siRNA to neuronal cells in the mouse brain (Alvarez-Erviti et al., 2011). Similarly, the integrin-recognition motif Arg-Gly-Asp (RGD), present on viral envelopes, mediates host cell infection (Hussein et al., 2015) by driving viral particle internalization and endosomal escape (Shayakhmetov et al., 2005). Incorporation of LAMP2 fused to RGD in the membrane shield of EVs results in an efficient EV delivery of the chemotherapeutic doxorubicin to integrin-positive breast cancer cells (Tian et al., 2014b). Lipids participate as well in membrane fusion events (Mathieu et al., 2019). For instance, PS on the EV surface may interact with the PS-ligand annexin-V enriched in membranes of early and late endocytic organelles of macrophages (Diakonova et al., 1997). In an opposite manner, PS on the luminal side of endocytic membranes (Matsudaira et al., 2017) binds to annexin-V present on the surface of apoptotic bodies (Igami et al., 2020). Cholesterol is a key component of membrane organization and fusion events (Yang et al., 2016). Indeed, the incorporation of cholesterol in EV membranes is tightly regulated, and the endocytic process largely depends on cholesterol (Mulcahy et al., 2014; Pfrieger and Vitale, 2018; Mathieu et al., 2019; Skotland et al., 2020). A recent report describes that treatment with U18666A, a ligand of Niemann–Pick C1 protein, causes endo-lysosomal accumulation of low-density lipoprotein–bound cholesterol and hampers EVs fusion with recipient cell membranes (Joshi et al., 2020). In a more provocative way, the description of an unconventional delivery of EV content directly into the nucleus of recipient cells mediated by late endosomes in contact with the nuclear envelope unveils a beforehand unexplored EV delivery mechanism (Rappa et al., 2017).

Independently of the mechanisms involved, the fusion of EVs with cell membranes is expected to result in the liberation of EV luminal cargos within the cytosol of the recipient cell. Several studies have exploited the extremely sensitive CRE-based recombination technique to infer on EV-mediated trans-cytosol transport of biologically active macromolecules. Strikingly, these studies reveal that cytosolic entry of an EV cargo occurs at a sporadic rate (Ridder et al., 2014, 2015; Zomer et al., 2015; Sterzenbach et al., 2017; Steenbeek et al., 2018; Ilahibaks et al., 2019), an observation in contradiction with the large body of evidence showing an efficient EV uptake through endocytosis.

## The Endo-Lysosomal System, a Crossroad for Exogenous and Endogenous Biomolecules?

The molecular mechanisms governing EV cargo loading dictate not only the specific cargo signature of an EV and thus the specific effect it will elicit in recipient cells, but also the preferential route of delivery (van Niel et al., 2018). Among all the identified biomolecules transported within the EV lumen (Colombo et al., 2014), nucleic acids and proteins have been shown to elicit a response in recipient cells (Valadi et al., 2007; Skog et al., 2008; Zomer et al., 2015). The target machinery able to translate a nucleic acid–encoded message is expected within the cytosol or the nucleus of recipient cells, with delivery requiring membrane fusion events. A different prospect applies for a protein cargo, as various surface and intracellular targets are apt for engagement, thus widening the possible location for the delivery of the message. Additional intracellular locations besides the cell surface and the cytosol, discussed above, are plausible. Recent studies imply an EV-mediated signaling in the endo-lysosomal compartment. Human mast cell–derived EVs promote phenotypic changes in recipient mesenchymal stem cells through transforming growth factor beta1 (TGFbeta1) signaling (Shelke et al., 2019). The role of endosomes in this signaling is demonstrated by requirement of an acidic pH for the activation of TGFB1 (Annes et al., 2003; Shelke et al., 2019). EVs loaded with the enzyme beta-glucocerebrosidase (GBA) cause increased lysosomal GBA activity in recipient cells to a similar extent when GBA was engineered in the lumen or on the surface of EVs (Do et al., 2019a). These data indicate that macromolecules transported through EVs could remain functionally active in organelles of the endo-lysosomal pathway. Endo-lysosome organelles represent a subcellular compartment where degradative pathways able to transfer exogenous and endogenous proteins converge (Lawrence and Zoncu, 2019). In other words, the endo-lysosomal compartment represents a crossroad where extracellular molecules delivered through “cell-eating” and “cell-drinking” endocytic pathway may meet intracellular molecules transported by the “self-eating” autophagic machinery (Mizushima and Komatsu, 2011).

## Self-Eating

**FIGURE 2:**
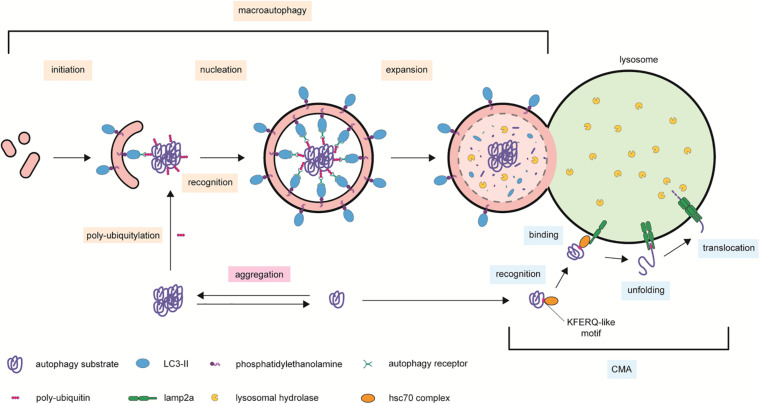
Autophagic processes contributing to cellular proteostasis. Protease-resistant protein complexes and aggregates are delivered to lysosomal degradation by macroautophagy. This process is mediated by scaffold autophagy receptors able to bind to ubiquitinated proteins and the cleaved and phosphatidylethanolamine-conjugated LC3 present on the forming autophagosome. The complex is then encapsulated within the lumen of the double-lipid by-layer autophagy organelle, which expands and fuses with lysosome membranes for acidic hydrolases degradation. In chaperon-mediated autophagy (CMA), cytosolic proteins bearing a KFERQ-like domain recognized by the unfolding chaperon HSC70 are escorted to LAMP2A on the surface of lysosomal membranes. Two consecutive recruitments of LAMP2A molecules form a module that translocates the CMA substrate to the hydrolase-rich lumen of lysosomes. Symbols used are specified in the legend on the bottom of the scheme.

Protein homeostasis is maintained in mammalian cells by multiple systems (Li et al., 2012). The ubiquitin (Ub)–proteasome system (UPS) is a selective proteolytic machinery, in which (usually) short-living poly-ubiquitinated proteins are unfolded and degraded by the proteasome (Mizushima and Komatsu, 2011). The term *autophagy* originates from the Greek words αùτó*ς* (auto = self) and φαγεîν (phagy = eating), hence describing a self-eating process (Wesselborg and Stork, 2015). The autophagic machinery is complex in terms of both distinct mechanisms and substrate heterogeneity; this latter spanning from (usually) long-living proteins, protein aggregates, nucleic acids, and cellular organelles (Mizushima and Komatsu, 2011; Fujiwara et al., 2017). Moreover, besides its degradative function, autophagy is a truly dynamic process that serves also as a recycling system providing the cell with the material required to maintain its energetic homeostasis (Mizushima and Komatsu, 2011). The broad term “autophagy” encompasses three main processes: macroautophagy, microautophagy, and chaperone-mediated autophagy (CMA) (He and Klionsky, 2009; Tooze and Yoshimori, 2010; Feng et al., 2014; Dikic and Elazar, 2018). Macroautophagy sequesters and encapsulates cytoplasmic components including whole organelles or organelle portions within an intermediate double-phospholipid-bilayer organelle named “autophagosome” (Xie and Klionsky, 2007; Tooze and Yoshimori, 2010; Lawrence and Zoncu, 2019). This latter travels along microtubules toward the perinuclear region where it fuses either directly with lysosomes or with late endosomes as an intermediate step (Xie and Klionsky, 2007; Tooze and Yoshimori, 2010; Mizushima and Komatsu, 2011; Nakamura and Yoshimori, 2017). In contrast, microautophagy sequesters small components of the cytoplasm (Mijaljica et al., 2011). Non-selective microautophagy is specific toward small cytosolic substrates, e.g., soluble proteins, and is characterized by the formation of tubular invaginations of the lysosomal membranes (Mijaljica et al., 2011; Li et al., 2012). These membrane invaginations pinch off, forming intralysosomal vesicles, which then release their cargo in the hydrolase-rich environment of lysosomes (Sahu et al., 2011). Inward membrane budding is also at the basis of ILV biogenesis in MVBs, a process that may be suitable for eliminating cytosolic components by secretion. On the other hand, selective microautophagy is specific toward large substrates or organelles (e.g., mitochondria, nucleus, and peroxisomes). These are engulfed through the projected, arm-like protrusion of lysosomal membranes; internalized within the lumen of lysosomes; and then gradually digested (Mijaljica et al., 2011; Mizushima and Komatsu, 2011; Li et al., 2012). Unlike the other two types of autophagy, in CMA the combined action of the chaperone protein heat shock cognate 70 (HSC70) and the lysosomes-associated membrane protein type 2A (LAMP2A) results in the specific recognition of protein substrates carrying a KFERQ-like pentapeptide and in their active translocation across lysosomal membranes (Mizushima et al., 2008; Orenstein and Cuervo, 2010; Mizushima and Komatsu, 2011; Mizushima, 2018; Figure 2).

Autophagy is thus a diversified system that continuously delivers a large variety of intracellular substrates to the endo-lysosomal compartments. In this way, it is a process that, at any given time, expands the array of macromolecules available to meet extracellular material internalized through endocytosis, with possible implications in health and disease. Notably, EV cargos such as mRNAs and miRNAs may directly regulate the autophagic processes once released within recipient cells (Song et al., 2016; Cai et al., 2017; Kulkarni et al., 2018). This encounter, within an acidic compartment dedicated to degradation, may gain relevance in cellular states where lysosomal activity is compromised, and substrates may accumulate.

## Autophagy–Lysosome Dysfunction in Neurodegenerative Diseases

**FIGURE 3:**
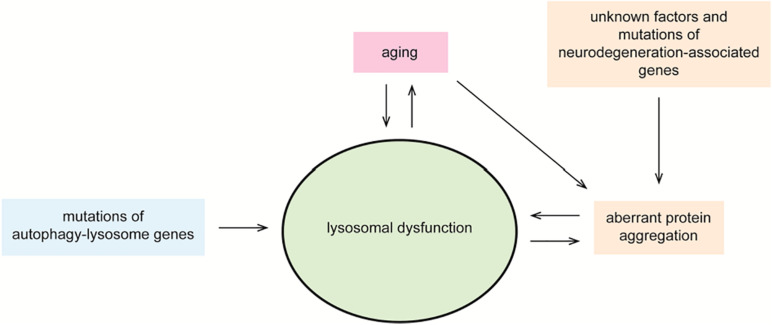
Lysosomal dysfunction in neurodegeneration. Controlled degradation of proteins via lysosomal hydrolases is a key cellular homeostatic event. Aging, unknown factors, mutations in neurodegeneration-associated genes and in autophagy–lysosome genes, and the accumulation of cytosolic protein inclusions are negative regulators of lysosomal function. In a vicious circle, defective lysosomal function contributes to aging, accumulation of toxic gene products, and disease. Impaired lysosomal function may occur at the level of acidic hydrolase activity or by altered fusion and maturation of autophagy–lysosome organelles.

Neurodegenerative disorders, such as AD, Parkinson disease (PD), Huntington disease (HD), amyotrophic lateral sclerosis (ALS), and transmissible spongiform encephalopathies (TSEs), are etiologically and clinically distinct. Crucially, they all share as pathological hallmark the deposition of protein aggregates into ubiquitinated intraneuronal inclusions (Brundin et al., 2010; Ciechanover and Kwon, 2015). Each disorder-specific protein aggregate is formed by distinct proteins, which acquire a beta-sheet–enriched conformation and eventually form soluble multimeric structures and insoluble protein inclusions. Beta-amyloid and tau are linked to AD (Terry, 1963; Masters et al., 1985; Grundke-Iqbal et al., 1986; Goate et al., 1991; Haass and Selkoe, 1993), alpha-synuclein to PD (Polymeropoulos et al., 1997; Uversky, 2007), huntingtin to HD (Davies et al., 1997; DiFiglia et al., 1997), TDP43 to ALS, and frontotemporal lobar degeneration (FTLD) (Arai et al., 2006; Neumann et al., 2006) and prion to TSE (Aguzzi, 2007). A recognized risk factor for this family of disorders is aging, as well as the gradual impairment of the cellular degradative systems (Kovács et al., 2007; Piras et al., 2016; Malik et al., 2019). Postmitotic neurons of the central nervous system are particularly vulnerable to the impairment of the autophagy–lysosome pathway. This age-dependent progressive deficiency observed in the aging brain correlates with the increasing accumulation of potentially toxic protein forms in the neurodegenerating brain (Wong and Cuervo, 2010; Lamark and Johansen, 2012; Ciechanover and Kwon, 2015; Metaxakis et al., 2018). This is further supported by animal models of late-onset neurodegenerative disorders, which display a progressive accumulation of autophagic organelles and protein aggregates (Spencer et al., 2009; Decressac et al., 2013; Valionyte et al., 2020). Furthermore, nanoscale analysis performed on postmortem brain tissue slice of patients affected by AD and PD showed that beta-amyloid and alpha-synuclein inclusions were enriched in lipid membranes and organelles structurally resembling lysosomes and in part immune-reactive for lysosomal markers (Nixon et al., 2005; Hassiotis et al., 2018; Shahmoradian et al., 2019). Similar observations were made with the identification of intralysosomal prion inclusions in neurons of sporadic Creutzfeldt–Jakob disease brains (Kovács et al., 2007). Actually, most studies demonstrated that cytosolic protein inclusions are substrates of selective macroautophagy and that molecular interventions aimed to stimulate macroautophagy reduce intraneuronal protein deposition with concomitant decrease in cell toxicity and amelioration in behavioral phenotypes in animal models (Tanaka et al., 2004; Spencer et al., 2009; Caccamo et al., 2010; Spilman et al., 2010; Yang et al., 2011; Steele et al., 2013; Savolainen et al., 2014; Yoon et al., 2017; Djajadikerta et al., 2019). Intuitively, a defect in a key clearance mechanism of the cell is expected to directly contribute to the build-up of aberrant proteins predestined to be eliminated. On the other hand, cellular inclusions and lysosomal overload may contribute to a vicious cycle of events curbing lysosomal dysfunction and protein deposition. Nevertheless, the discussion whether defective activity of the autophagy–lysosome pathway is a “cause” or an “effect” of intracellular protein inclusions merits more attention (Figure 3).

### The “Cause”

Compelling genetic and pharmacological experimental evidence supports that impairment of the autophagy–lysosome pathway exacerbates the accumulation of potentially toxic protein oligomers and causes neurodegeneration. To reinforce this argument, an approach often used is to abolish or reduce the transcription of genes belonging to the core machinery of autophagy. The autophagy-related 5 (*ATG5*) gene encodes for a protein that conjugates with ATG12 and ATG16 to form a complex involved in the extension of autophagosome membranes (Tanida, 2011). Mice lacking Atg5 expression specifically in neurons are characterized by the progressive neuronal accumulation of cytoplasmic, Ub-positive inclusion bodies and by the concomitant progressive deficit in motor and behavioral functions (Hara et al., 2006). ATG7 is an E1-like activating enzyme whose primarily function is to participate in the conjugation of ATG12 and in the lipidation of the microtubule-associated protein light chain 3 (LC3), two essential steps in the formation of functional autophagosomes (Xiong, 2015). The study of *Atg7* knockout mice provides additional evidence for the contribution of autophagy in the formation of protein inclusions with aberrant intraneuronal accumulation of beta-amyloid and cognitive dysfunction in a mouse model of AD (Nilsson et al., 2013). Beclin-1, another regulator of macroautophagy, appears altered in aged brains and in patients affected by AD and HD. Decreased beclin-1 in mice models of AD- and HD-related amyloidosis causes impaired macroautophagy, increased inclusion bodies, and general neuronal deficits, aspects reversed through ectopic beclin-1 expression (Shibata et al., 2006; Pickford et al., 2008; Lucin et al., 2013).

To date, there is only a single report of a human pathogenic mutation among all the core *ATG* genes. The homozygous E122D mutation in *ATG5* was found in two siblings affected by a childhood form of ataxia, characterized by progressive loss of Purkinje cells, cerebellar hypotrophy, and clinical symptoms affecting muscle coordination (Kim et al., 2016). At the cellular level, the single point mutation leads to impaired autophagy flux caused by defective conjugation of ATG5 with ATG12 (Kim et al., 2016).

Selective macroautophagy depends on Ub-binding scaffold proteins that recognize cytoplasmic ubiquitinated protein substrates and deliver them to the autophagy pathway for degradation (Zientara-Rytter and Subramani, 2019). Among all, P62/sequestome 1 (encoded by the *SQSTM1* gene), NBR1 autophagy receptor (*NBR1*, neighbor of *BRCA1* gene), autophagy-linked FYVE protein ALFY (*WDFY3*), and optineurin (*OPTN*) are found in almost all types of protein aggregates (Pankiv et al., 2007; Stolz, 2014; Lim and Yue, 2015). The presence of autophagy receptors within cytoplasmic inclusions supports the view that protein aggregates are cleared by a selective macroautophagic process (aggrephagy) (Øverbye, 2007). Concomitantly, the presence of autophagy receptors within cytoplasmic inclusions is also linked to a role of these as facilitators of protein aggregation (Pankiv et al., 2007; Shen et al., 2015). Most autophagy receptors are scaffold proteins that carry an Ub-associated (UBA) domain and an LC3-interacting region (LIR). The UBA domain binds to mono-ubiquitinated and poly-ubiquitinated proteins (Shaid et al., 2013; Stolz, 2014; Deng et al., 2017). On the other hand, the LIR sequence binds to LC3 conjugated to the inner surface of the phagophore (LC3-II), thus mediating the encapsulation of the complex in autophagosomes (Stolz, 2014; Deng et al., 2017; Dikic and Elazar, 2018), whereby autophagy receptors and LC3-II become themselves autophagy substrates (Deng et al., 2017). Genetic variants of the autophagy receptors *OPTN* and *SQSTM1* are linked to ALS-FTLD (Fecto et al., 2011; Deng et al., 2017), and more severe disease forms are caused by mutations in the UBA domain of P62 (Fecto et al., 2011; Kwok et al., 2014). Furthermore, P62, optineurin, and NBR1 localize within Lewy bodies and neurofibrillary tangles (NFTs) in postmortem human brain tissue (Osawa et al., 2011; Odagiri et al., 2012). At the cellular level, P62 binds to poly-ubiquitinated tau mediating its clearance, and mice with genetic *Sqstm1* inactivation display intraneuronal tau aggregation (Ramesh Babu et al., 2008).

Inherited mutations of lysosomal hydrolases are linked to neurodegenerative disorders (Lwin et al., 2004; Osellame et al., 2013; Ebrahimi-Fakhari et al., 2014; Menzies et al., 2017; Do et al., 2019b). For instance, homozygous mutation in the *GBA* gene encoding for the lysosomal enzyme glucocerebrosidase causes Gaucher disease, a lysosomal storage disorder (Mazulli et al., 2011; Menzies et al., 2017; Do et al., 2019b). Loss of GBA function triggers the accumulation of its substrate glucocerebroside within lysosomes leading to dysfunctional lysosomal degradation and autophagic processes (Mazulli et al., 2011; Menzies et al., 2017; Do et al., 2019b). Experimental *Gba* knockout mouse model of Gaucher disease displays defective autophagy with P62 accumulation, Ub-positive proteins, and oligomeric alpha-synuclein (Osellame et al., 2013). Notably, heterozygous mutation in *GBA* is the most common genetic risk factor for PD (Lwin et al., 2004; Menzies et al., 2017; Do et al., 2019b).

Chemical compounds are also used to mimic autophagy dysfunction and its consequences. Bafilomycin A1, a macrolide derived from *Streptomyces griseus*, specifically inhibits the vacuolar ATPase that transports protons to the interior of acidic organelles (Mauvezin and Neufeld, 2015). Chloroquine (CQ), a drug known for its antimalarial and anti-inflammatory properties, is a lysosomotropic buffering agent rapidly penetrating across cell membranes and undergoing a protonation-based trapping in the acidic environment of autophagic, endocytic, or lysosomal organelles (Al-Bari, 2015). The presence of either compound efficiently neutralizes the luminal pH, inhibits acidic hydrolases, and impairs the fusion among acidic organelles (Yamamoto et al., 1998; Mauvezin and Neufeld, 2015; Mauthe et al., 2018). Increased accumulation of cytosolic aggregates is found when these drugs are applied to *in vitro* and *in vivo* models of neurodegeneration. This occurs, for instance, in COS-7 cells expressing an aggregation-prone fragment of mutant huntingtin, where treatment with bafilomycin A1 results in a more pronounced aggregation determined by the increase in aggregates size and in the number of affected cells (Ravikumar et al., 2002). The same treatment exacerbates the formation of detergent-insoluble alpha-synuclein species in rat embryonic cortical neurons (Lee et al., 2004). An increment in seeding events is observed in primary neurons derived from tau transgenic mice when incubated with exogenous tau fibrils and CQ (Gibbons et al., 2017). A higher Ub-positive cytoplasmic TDP43 inclusion load is observed upon CQ treatment of a mouse model expressing mutated vasolin-containing protein (Custer et al., 2010; Nalbandian et al., 2015). In good agreement with these findings, macroautophagy stimulation is a proven intervention apt to reduce cytosolic protein aggregates, ultimately reversing behavioral phenotypes in animal models of neurodegenerative diseases (Ravikumar et al., 2004; Tanaka et al., 2004; Spencer et al., 2009; Caccamo et al., 2010; Spilman et al., 2010; Yang et al., 2011; Steele et al., 2013; Savolainen et al., 2014; Yoon et al., 2017; Djajadikerta et al., 2019). In a mouse model of beta-amyloid and tau pathology, induction of autophagy with an inhibitor of the mammalian target of rapamycin (mTOR) decreases intraneuronal beta-amyloid accumulation and rescues cognitive deficits (Caccamo et al., 2010). Similarly, oral administration of the disaccharide trehalose in a transgenic mouse model for mutant huntingtin effectively reduces cytosolic inclusions and ameliorates hallmark motor dysfunctions of HD (Tanaka et al., 2004). The neuroprotective effect of trehalose may rely on stimulation of autophagic flux independently from the mTOR signaling pathway (Sarkar et al., 2007; Lee et al., 2018).

### The “Effect”

The increased impairment of the autophagy–lysosome pathway associated with the progression of neurodegenerative disorders may hint to intrinsic negative effects caused by accumulating aberrant protein forms. This outcome may result from a loss of function; e.g., the scaffold protein huntingtin interacts with P62 and facilitates the association of ubiquitinated substrates targeted to autophagy with LC3 (Ochaba et al., 2014). Mutant huntingtin fails in this role, thus compromising cytosolic cargo recognition and delivery to selective autophagy (Martinez-Vicente et al., 2010).

UPS and CMA are the first-line defense in disposing soluble proteins. However, when proteins aggregate into fibrillar insoluble forms, as it is the case for neurodegenerative disorders, they become increasingly resistant to both UPS and CMA degradation (Ciechanover and Kwon, 2015). The capacity to “escape” UPS and CMA degradation may well rely on the resilience of structured, beta-sheet–enriched protein aggregates to chaperon-mediated “unfolding,” which is required for funneling the polypeptide into these degradative pathways. As a result, they may act as negative regulators. CMA-mediated degradation relies on the presence of the KFERQ-like motif on its substrates. Accordingly, the VKKDQ sequence on alpha-synuclein is a recognition and binding domain for the chaperon HSC70, which mediates its translocation into lysosomes (Fred Dice, 1990; Cuervo et al., 2004). Fibrillar forms of alpha-synuclein resist to the unfolding activity of HSC70, and by binding to LAMP2A, they act as translocation inhibitors further busting their accumulation and impairing the degradation of other CMA substrates (Cuervo et al., 2004; Ross and Poirier, 2005). Likewise, at least *in vitro*, cytosolic protein inclusions act as clogging blockers in the barrel-shaped structure of the proteasome (Cuervo et al., 2004; Martinez-Vicente et al., 2008; Andre and Tabrizi, 2012). However, UPS and CMA impairment stimulates the specialized activity of aggrephagy as an alternative degradative machinery (Massey et al., 2006; Pandey et al., 2007; Lamark and Johansen, 2012).

Interestingly, prion infection disrupts the maturation of endo-lysosomal organelles by interfering with RAB7 association to membranes, which eventually prevents lysosomal degradation of PrP^Sc^ in favor of fibril formation (Shim et al., 2016). The key molecular spark that triggers prion pathogenesis is the conformational conversion of prion protein PrP^C^, with a predominant alpha-helix content, into the highly infectious beta-sheet–rich PrP^Sc^ (Pan et al., 1993). PrP^Sc^ forms detergent-insoluble fibrils also defined as PrP^Res^ to highlight their intrinsic ability to resist to proteolytic degradation. As the endo-lysosomal pathway may represent the subcellular site where the conversion to PrP^Sc^ occurs, this has the potential to impair overall lysosomal degradative function (Caughey et al., 1991). Indeed, increased number and size of lysosomes and autophagic vacuoles are well-established neuropathological features of prion-infected neurons in animal models and in patients (Boellaard et al., 1991; Sikorska et al., 2004).

In addition to the effect of cytosolic aberrant protein forms, also their extracellular counterparts need to be considered when studying the pathogenesis of diseases. As previously discussed, non–cell-autonomous proteins exploit the endocytic pathway to access the inside of cells. In this respect, recombinant and brain-extracted protein fibrils and seeds enter the cell and traffic toward lysosomes upon endocytosis (Hu et al., 2009; Domert et al., 2016; Karpowicz et al., 2017; Evans et al., 2018), whereby lysosomal function appears required prior to reach the cytosol for further propagation (Tsujimura et al., 2015; Domert et al., 2016; Evans et al., 2018; Hoffmann et al., 2019). Intriguingly, exogenous alpha-synuclein fibrils taken up by endocytosis drive intracellular seeding within the endo-lysosomal compartment (Tsujimura et al., 2015). This occurs with the assistance of the lysosomal protease cathepsin-beta that, when compared other proteases, specifically triggers its aggregation. Concurrently, the accumulation of protease resistant alpha-synuclein fibrils within lysosomes impairs lysosomal function and autophagic flux (Hoffmann et al., 2019).

#### Protein Aggregation Within Lysosomes

**FIGURE 4:**
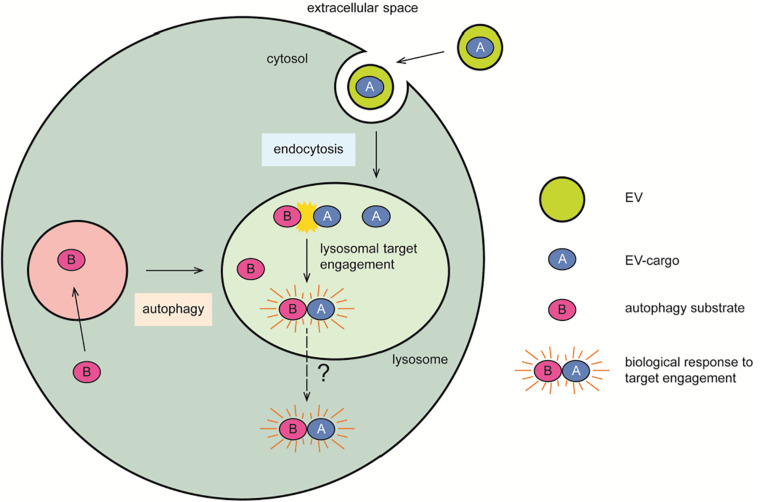
Lysosomal contribution to seeded propagation of disease. The endocytic organelles represent a crossroad where exogenous proteins (A) transported by EVs engage with their cellular cytosolic targets (B) transported by autophagy. The slightly acidic milieu of these organelles may spare proteins from degradation in favor of a biologically relevant target engagement, or of a pathogenic mechanism of seeded propagation of toxic protein forms. This latter may subsequently lead to lysosomal membrane rupture, overt cytotoxicity, and the formation of cytosolic protein inclusions as pathological hallmarks of disease. Symbols used are specified in the legend on the right of the scheme.

In the context of this review, it is intriguing to consider alternative intracellular locations besides the cytosol, which may serve as seeding hubs in the nucleation process leading to intraneuronal inclusions as observed in neurodegenerative disorders. The acidic organelles of the cell may represent the initial location for the seeding activity of exogenous protein oligomers and fibrils, which may then gain access to the cytosol and trigger further aggregation (Figure 4). The endo-lysosomal pathway of the cell gathers at least four features that ideally facilitate the mechanism of disease-causing protein aggregation: (1) the presence of proteases, (2) the co-assistance of other protein-modifying enzymes such as glycosidases, sulfatases, and kinases, (3) a low pH, and (4) a relatively small volume and membrane surface area.

Proteolytic processing of neurodegeneration-associated proteins has received particular attention as *in vitro* evidence suggests increased propensity to aggregate when these are cleaved at specific amino acid sites. We discussed already above the evidence that in a cellular model of prion infection, the amino terminus of PrP^Res^, or of its precursor, is removed by lysosomal proteases facilitating its aggregation within lysosomes (Caughey et al., 1991). It was recently reported that tau is a substrate of asparagine endopeptidase, a lysosomal cysteine protease generating tau fragments with a high propensity to aggregate (Chen et al., 1998) observed also in the brain of human AD or tau transgenic mice (Zhang et al., 2014). What is more, the release of beta-amyloid from its precursor is initiated by the activity of the acidic endoprotease BACE located in endo-lysosomes (Pasternak et al., 2004). Intriguingly, the discovery within lysosomes of kinases phosphorylating neurodegenerative-associated proteins has raised awareness that pathological phosphorylation may be acquired within these organelles. As an example, the beta-isoform of glycogen synthase kinase 3 (GSK3beta), the main phosphorylating enzyme for a tau form enriched in disease-associated NFTs (Cohen and Goedert, 2004), is found within acidic organelles of the endo-lysosome pathway (Taelman et al., 2010; Li et al., 2016). Although experimental evidence that directly links tau toxicity to lysosomal GSK3beta is missing, this mechanism is intriguing and cannot be disregarded.

A high load of energy is required for the activity of the proton pump to maintain the acidic conditions required for the activity of lysosomal enzymes (Mindell, 2012). A low pH could serve as a spark triggering protein aggregation as it was shown for prion conversion (DeMarco and Daggett, 2007; Srivastava and Lapidus, 2017). Indeed, a protonation-based model approach demonstrated that the partial unfolding and dissociation of one alpha-helices of PrP^C^ result in the loss of critical long-range salt bridges, which favor the conversion to a PrP^Sc^-like structure (DeMarco and Daggett, 2007). Similar results were obtained analyzing kinetics of amyloid fibril formation, which is accelerated in the pH range observed in lysosomes of living cells, implying a possible contribution of lysosomes in amyloid diseases (Colon and Kelly, 1992).

The relatively small size of endo-lysosomal organelle when compared to the cytosol should also be considered a critical contributor of neurotoxic protein aggregation. Seeding aggregation of proteins is remarkably dependent on protein concentration and on the interaction with membranes (Eisele et al., 2015; Poulson et al., 2020), whereby a threshold concentration has to be reached in order to initiate a nucleation-dependent polymerization (Harper and Lansbury, 1997). Autosomal dominant disease forms bring clinical evidence of the correlation between protein concentration and pathogenicity. Examples are a hereditary PD form characterized by the triplication of the alpha-synuclein locus and extensive Lewy body formation (Singleton et al., 2003) or trisomy 21 characterized by an extra copy of the *APP* gene and early-onset AD-like amyloidosis (Lott and Head, 2019). *In vitro* experiments support the view that lysosomes may foster the critical concentration and exposure to membranes required for protein nucleation and further multimerization. This is the case when extracellular beta-amyloid monomers were found to be taken up by cells and to accumulate with seeding nucleation properties within lysosomes (Hu et al., 2009).

#### Disease Spreading and EVs

Neurodegenerative diseases are characterized by the spreading of pathological protein forms following a predictable spatiotemporal pattern through the brain of affected patients. This correlates with the symptom progression in a disorder-specific and unique manner. For instance, in AD, tau NFTs first occur in the entorhinal region and then spread to the surrounding hippocampal area and reach the entire neocortex in later disease stages (Goedert, 2015; Kaufman et al., 2018). In contrast, beta-amyloid senile plaques are first observed in the orbitofrontal and basal temporal neocortex and then slowly progress from anterior to posterior areas to invade the entire neocortex, the hippocampus, the amygdala, and the basal ganglia (Goedert, 2015). At the cellular level, nucleation-competent seeds are transferred from cell-to-cell, possibly exploiting existing cell communication mechanisms to drive the spreading of pathology. At the molecular level, the conversion of a native (often unfolded) state to a highly ordered fibrillar structure resembles the template-mediated mechanisms of prions, thus defined as prion-like paradigm (Soto and Pritzkow, 2018). *In vitro* studies support the notion that the dissemination of pathogenic protein forms through templated amplification occurs via interconnected neurons (Gribaudo et al., 2019; Hallinan et al., 2019), which fits well the progressive spreading through anatomically linked brain regions observed *in vivo* (Narasimhan et al., 2017; Henderson et al., 2019; Wegmann et al., 2019). Noteworthy, some connected brain areas are spared by this process, hinting to a specific cellular mechanism of release/uptake of protein seeds. Among the non-exclusive routes of cell-to-cell seed transport, EVs have gained a significant recognition as likely transcellular vehicles (Fiandaca et al., 2015; Thompson et al., 2016; Guix et al., 2018), with the advantage to ensure protection against degradative activities and propagation to distant targets. This pathogenic role of EVs is supported by observations made in animal models of most neurodegenerative disorders (Vella et al., 2007; Watson et al., 2019). Not surprisingly, there is the demand to better define whether a specific mechanism exists for the encapsulation of nucleation-competent particles into EVs by the (infectious) donor cell. At the same time, and possibly more importantly, we need to understand how seeds are internalized by the (healthy) recipient cell to reach their target. As discussed above, EVs mainly take advantage of the endocytic pathway to enter the cell where they may accumulate within lysosomes and liberate their possibly noxious cargo. Accordingly, a recent *in vitro* study from our laboratory brought evidence that it is conceivable that an EV-transported, proaggregating tau form uses this route of cell entry to eventually induce cellular tau accumulation within acidic organelles of the recipient cell. The physical interaction between exogenous seeds and endogenous wild-type tau at the crossroad between the endocytic and the autophagic pathways ultimately triggered the formation of tau epitopes typical of NFTs, progressive lysosomal impairment, and overt cytotoxicity (Pedrioli et al., 2020). Thus, despite the dichotomy of degradative organelles embodying the cellular site where seeded propagation of pathogenic protein occurs, mounting experimental evidence points to a role of EVs as transcellular mediators exacerbating an (age-related) impairment of the lysosomal pathway in the neurodegenerative state.

## Conclusion and Future Directions

A constantly growing knowledge on the biology of EVs has expanded the initial, but still valid, interpretation of them as a kind of garbage bag expelled by the cell, to include now for them a role as vehicle of precise cell-to-cell communication and as critical contributor to disease. Technologic advances allowed a detailed, yet in part incomplete, perception of the complexity of this variegate population of secreted vesicles. A main objective of this review is to discuss the cellular mechanisms required to elicit a biological response in the acceptor cell, while much is already known in terms of EV biogenesis in the donor cell. Among them, the list includes the specific tropism toward the recipient cell, the route evolved for their uptake, and the release mechanism for the target engagement of a biological active cargo molecule. Distinct subpopulations of EVs, their cargo signatures, and the type and state of recipient cells, to name a few, are all puzzle fragments contributing to the complexity of the picture currently assembled.

This review has focused on the experimental evidence pointing to the central role for EV-mediated cellular communication provided by the endocytic pathway. In an organism, cells are exposed, at any given time, to EVs continuously released from a wide range of cell populations, with distinct biophysical properties and cargo compositions and originating from close and distant locations. Some questions relating to the mechanisms regulating the tropism of EVs remain partially unanswered. In this plethora of EVs floating on the surrounding of a cell, how is a message delivered with accurate precision to the desired target cell? Does the endocytic pathway merely represent a route exploited by EVs to gain access to the cell, or does it provide the favorable environment for functional cargo delivery in health and disease? Will the knowledge on the biology driving viral infection of a cell facilitate the identification of the mechanisms governing EV cargo release, or do we need to implement innovative and highly sensitive research methods for this purpose? The current techniques suggest that release of an EV cargo in the cytosol of recipient cells is an existing but rather rare event. An important question to be addressed will be to define “rare” in the context of a biologically relevant EV-mediated cell-to-cell communication.

Finally, we also discussed the existence of a possible vicious cycle driving a neurodegenerative process (Figure 3). Aggregation of aberrant proteins may impair autophagic and lysosomal degradative pathways, which in turn curb further protein aggregation, and importantly seeded transcellular propagation possibly mediated by EVs as vectors for direct delivery of replication-competent particles to acidic organelles of recipient cells. Even more, EVs could be the trigger for the initiation of a cascade of adverse events including prion-like propagation and lysosomal dysfunction. In this context, autophagy stimulation as a proposed intervention to reduce intraneuronal protein inclusions may backfire into a completely opposite direction. If not specifically targeted to the affected neuron, autophagy stimulation may well favor that an EV cargo with seeding capabilities could encounter and propagate on the native protein counterpart within lysosomes of healthy neurons. The potential dichotomic role of the autophagy–lysosome pathway in clearing cytosolic inclusions and contributing to transcellular propagation certainly requires further attention and experimental validation.

## Author Contributions

GP and PP wrote, reviewed, and edited the original draft. Both authors contributed to the article and approved the submitted version.

## Conflict of Interest

The authors declare that the research was conducted in the absence of any commercial or financial relationships that could be construed as a potential conflict of interest.

## Abbreviations

AD: Alzheimer disease
AEP: asparagine endopeptidase
ALS: amyotrophic lateral sclerosis
AP2: adaptor protein 2
ATG: autophagy-related
CAME: caveolin-mediated endocytosis
CLME: clathrin-mediated endocytosis
CMA: chaperone-mediated autophagy
CQ: chloroquine
EIPA: 5-(N-ethyl-N-isopropyl)amirolide
ESCRT: endosomal sorting complex required for transport
EVs: extracellular vesicles
FTLD: frontotemporal lobar degeneration
GBA: beta-glucocerebrosidase
GSK3beta: glycogen synthase kinase 3 beta
HD: Huntington disease
HSC70: chaperone protein heat shock cognate 70
ILVs: intraluminal vesicles
LAMP2A: lysosomes-associated membrane protein type 2A
LC3: microtubule-associated protein light chain 3
LIR: LC3-interacting region
MCL: mantle cell lymphoma
MHC-II: major histocompatibility complex II
mTOR: mammalian target of rapamycin
MVBs: multivesicular bodies
NFTs: neurofibrillary tangles
NHE: Na^+^/H^+^ exchanger
TGFbeta1: transforming growth factor beta1
PD: Parkinson disease
PS: phosphatidylserine
RGD: Arg-Gly-Asp
RVG: rabies virus glycoprotein
TSE: transmissible spongiform encephalopathies
UBA: ubiquitin-associated
UPS: ubiquitin-proteasome system.

## References

[B1] Aguzzi M. (2007). Heikenwalder, and M. Polymenidou, Insights into prion strains and neurotoxicity. *Nat. Rev. Mol. Cell Biol.* 8 552–561. 10.1038/nrm2204 17585315

[B2] Al-Bari M. A. (2015). Chloroquine analogues in drug discovery: new directions of uses, mechanisms of actions and toxic manifestations from malaria to multifarious diseases. *J. Antimicrob. Chemother.* 70 1608–1621. 10.1093/jac/dkv018 25693996 PMC7537707

[B3] Alvarez-Erviti L. Seow Y. Yin H. Betts C. Lakhal S. Wood M. J. A. (2011). Delivery of siRNA to the mouse brain by systemic injection of targeted exosomes. *Nat. Biotechnol.* 29 341–345. 10.1038/nbt.1807 21423189

[B4] Andre R. Tabrizi S. J. (2012). Misfolded PrP and a novel mechanism of proteasome inhibition. *Prion* 6 32–36. 10.4161/pri.6.1.18272 22453175 PMC3338962

[B5] Annes J. P. Munger J. S. Rifkin D. B. (2003). Making sense of latent TGFbeta activation. *J. Cell Sci.* 116 217–224. 10.1242/jcs.00229 12482908

[B6] Arai T. Hasegawa M. Akiyama H. Ikeda K. Nonaka T. Mori H. (2006). TDP-43 is a component of ubiquitin-positive tau-negative inclusionsin frontotemporal lobar degeneration and amyotrophic lateral sclerosis. *Biochem. Biophys. Res. Commun.* 351 602–611. 10.1016/j.bbrc.2006.10.093 17084815

[B7] Araki N. Johnson M. T. Swanson J. A. (1996). A role for phosphoinositide 3-kinase in the completion of macropinocytosis and phagocytosis by macrophages. *J. Cell Biol.* 135 1249–1260. 10.1083/jcb.135.5.1249 8947549 PMC2121091

[B8] Banchereau J. Steinman R. M. (1998). Dendritic cells and the control of immunity. *Nature* 392 245–252. 10.1038/32588 9521319

[B9] Bloomfield G. Kay R. R. (2016). Uses and abuses of macropinocytosis. *J. Cell Sci.* 129 2697–2705. 10.1242/jcs.176149 27352861

[B10] Boellaard J. W. Kao M. Schlote W. Diringer H. (1991). Neuronal autophagy in experimental scrapie. *Acta Neuropathol.* 82 225–228. 10.1007/bf00294449 1927279

[B11] Brundin P. Melki R. Kopito R. (2010). Prion-like transmission of protein aggregates in neurodegenerative diseases. *Nat. Rev. Mol. Cell Biol.* 11 301–307. 10.1038/nrm2873 20308987 PMC2892479

[B12] Bryant D. M. Kerr M. C. Hammond L. A. Joseph S. R. Mostov K. E. Teasdale R. D. (2007). EGF induces macropinocytosis and SNX1-modulated recycling of E-cadherin. *J. Cell Sci.* 120 1818–1828. 10.1242/jcs.000653 17502486

[B13] Burkard C. Verheije M. H. Wicht O. Van Kasteren S. I. Van Kuppeveld F. J. Haagmans B. L. (2014). Coronavirus cell entry occurs through the endo-/lysosomal pathway in a proteolysis-dependent manner. *PLoS Pathog.* 10:e1004502. 10.1371/journal.ppat.1004502 25375324 PMC4223067

[B14] Caccamo A. Majumder S. Richardson A. Strong R. Oddo S. (2010). Molecular interplay between mammalian target of rapamycin (mTOR), Amyloid-β, and Tau. *J. Biol. Chem.* 285 13107–13120. 10.1074/jbc.m110.100420 20178983 PMC2857107

[B15] Cai S. Shi G.-S. Cheng H.-Y. Zeng Y.-N. Li G. Zhang M. (2017). Exosomal miR-7 mediates bystander autophagy in lung after focal brain irradiation in mice. *Int. J. Biol. Sci.* 13 1287–1296. 10.7150/ijbs.18890 29104495 PMC5666527

[B16] Canton J. (2018). Macropinocytosis: new insights into its underappreciated role in innate immune cell surveillance. *Front. Immunol.* 9:2286. 10.3389/fimmu.2018.02286 30333835 PMC6176211

[B17] Caughey B. Raymond G. Ernst D. Race R. (1991). N-terminal truncation of the scrapie-associated form of PrP by lysosomal protease(s): implications regarding the site of conversion of PrP to the protease-resistant state. *J. Virol.* 65 6597–6603. 10.1128/jvi.65.12.6597-6603.1991 1682507 PMC250721

[B18] Chen J. M. Rawlings N. D. Stevens R. A. Barrett A. J. (1998). Identification of the active site of legumain links it to caspases, clostripain and gingipains in a new clan of cysteine endopeptidases. *FEBS Lett.* 441 361–365. 10.1016/s0014-5793(98)01574-9 9891971

[B19] Chiba M. Kubota S. Sato K. Monzen S. (2018). Exosomes released from pancreatic cancer cells enhance angiogenic activities via dynamin-dependent endocytosis in endothelial cells *in vitro*. *Sci. Rep.* 8:11972. 10.1038/s41598-018-30446-1 PMC6086824 30097593

[B20] Ciechanover A. Kwon Y. T. (2015). Degradation of misfolded proteins in neurodegenerative diseases: therapeutic targets and strategies. *Exp. Mol. Med.* 47:e147. 10.1038/emm.2014.117 25766616 PMC4351408

[B21] Cocucci E. Meldolesi J. (2015). Ectosomes and exosomes: shedding the confusion between extracellular vesicles. *Trends Cell Biol.* 25 364–372. 10.1016/j.tcb.2015.01.004 25683921

[B22] Cohen P. Goedert M. (2004). GSK3 inhibitors: development and therapeutic potential. *Nat. Rev. Drug Discov.* 3 479–487. 15173837 10.1038/nrd1415

[B23] Colin M. Delporte C. Janky R. S. Lechon A.-S. Renard G. Van Antwerpen P. (2019). Dysregulation of macropinocytosis processes in glioblastomas may be exploited to increase intracellular anti-cancer drug levels: the example of temozolomide. *Cancers* 11:411. 10.3390/cancers11030411 30909495 PMC6468498

[B24] Colombo M. Raposo G. Thery C. (2014). Biogenesis, secretion, and intercellular interactions of exosomes and other extracellular vesicles. *Annu. Rev. Cell Dev. Biol.* 30 255–289. 10.1146/annurev-cellbio-101512-122326 25288114

[B25] Colon W. Kelly J. W. (1992). Partial denaturation of transthyretin is sufficient for amyloid fibril formation in vitro. *Biochemistry* 31 8654–8660. 10.1021/bi00151a036 1390650

[B26] Costa Verdera H. Gitz-Francois J. J. Schiffelers R. M. Vader P. (2017). Cellular uptake of extracellular vesicles is mediated by clathrin-independent endocytosis and macropinocytosis. *J. Control. Release* 266 100–108. 10.1016/j.jconrel.2017.09.019 28919558

[B27] Cuervo A. M. Stefanis L. Fredenburg R. Lansbury P. T. Sulzer D. (2004). Impaired degradation of mutant a-synuclein by chaperone-mediated autophagy. *Science* 305 1292–1295. 10.1126/science.1101738 15333840

[B28] Custer S. K. Neumann M. Lu H. Wright A. C. Taylor J. P. (2010). Transgenic mice expressing mutant forms VCP/p97 recapitulate the full spectrum of IBMPFD including degeneration in muscle, brain and bone. *Hum. Mol. Genet.* 19 1741–1755. 10.1093/hmg/ddq050 20147319

[B29] Davies S. W. Turmaine M. Cozens B. A. Difiglia M. Sharp A. H. Ross C. A. (1997). Formation of neuronal intranuclear inclusions underlies the neurological dysfunction in mice Transgenic for the HD Mutation. *Cell* 90 537–548. 10.1016/s0092-8674(00)80513-9 9267033

[B30] de Oliveira C. A. Mantovani B. (1988). Latrunculin A is a potent inhibitor of phagocytosis by macrophages. *Life Sci.* 43 1825–1830. 10.1016/0024-3205(88)90282-2 3200109

[B31] Decressac M. Mattsson B. Weikop P. Lundblad M. Jakobsson J. Bjorklund A. (2013). TFEB-mediated autophagy rescues midbrain dopamine neurons from a-synuclein toxicity. *Proc. Natl. Acad. Sci.* 110 E1817–E1826. 23610405 10.1073/pnas.1305623110 PMC3651458

[B32] DeMarco M. L. Daggett V. (2007). Molecular Mechanism for Low pH triggered misfolding of the human prion protein. *Biochemistry* 46 3045–3054. 10.1021/bi0619066 17315950

[B33] Deng Z. Purtell K. Lachance V. Wold M. S. Chen S. Yue Z. (2017). Autophagy receptors and neurodegenerative diseases. *Trends Cell Biol.* 27 491–504. 10.1016/j.tcb.2017.01.001 28169082

[B34] Diakonova M. Gerke V. Ernst J. Liautard J.-P. van der Vusse G. Griffiths G. (1997). Localization of five annexins in J774 macrophages and on isolatedphagosomes. *J. Cell Sci.* 110 1199–1213. 9191044 10.1242/jcs.110.10.1199

[B35] DiFiglia M. Sapp E. Chase K. O. Davies S. W. Bates G. P. Vonsattel J. P. (1997). Aggregation of huntingtin in neuronal intranuclear inclusions and dystrophic neurites in brain. *Science* 277 1990–1993. 10.1126/science.277.5334.1990 9302293

[B36] Dikic I. Elazar Z. (2018). Mechanism and medical implications of mammalian autophagy. *Nat. Rev. Mol. Cell Biol.* 19 349–364. 10.1038/s41580-018-0003-4 29618831

[B37] Djajadikerta A. Keshri S. Pavel M. Prestil R. Ryan L. Rubinsztein D. C. (2019). Autophagy induction as a therapeutic strategy for neurodegenerative diseases. *J. Mol. Biol.* 432 2799–2821. 10.1016/j.jmb.2019.12.035 31887286

[B38] Do J. McKinney C. Sharma P. Sidransky E. (2019b). Glucocerebrosidase and its relevance to Parkinson disease. *Mol. Neurodegener.* 14:36. 10.1186/s13024-019-0336-2 PMC6716912 31464647

[B39] Do M. A. Levy D. Brown A. Marriott G. Lu B. (2019a). Targeted delivery of lysosomal enzymes to the endocytic compartment in human cells using engineered extracellular vesicles. *Sci. Rep.* 9:17274. 10.1038/s41598-019-53844-5 PMC6872767 31754156

[B40] Domert J. Sackmann C. Severinsson E. Agholme L. Bergström J. Ingelsson M. (2016). Aggregated Alpha-Synuclein transfer efficiently between cultured human neuron-like cells and localize to lysosomes. *PLoS One* 11:e0168700. 10.1371/journal.pone.0168700 28030591 PMC5193351

[B41] Durak-Kozica M. Baster Z. Kubat K. Stêpień E. (2018). 3D visualization of extracellular vesicle uptake by endothelial cells. *Cell. Mol. Biol. Lett.* 23:57. 10.1186/s11658-018-0123-z PMC6296015 30574165

[B42] Dusoswa S. A. Horrevorts S. K. Ambrosini M. Kalay H. Paauw N. J. Nieuwland R. (2019). Glycan modification of glioblastoma-derived extracellular vesicles enhances receptor-mediated targeting of dendritic cells. *J. Extracell. Vesicles* 8:1648995. 10.1080/20013078.2019.1648995 31489145 PMC6713149

[B43] Dutta D. Williamson C. D. Cole N. B. Donaldson J. G. (2012). Pitstop 2 Is a potent inhibitor of clathrin-independent endocytosis. *PLoS One* 7:e45799. 10.1371/journal.pone.0045799 23029248 PMC3448704

[B44] Ebrahimi-Fakhari D. Wahlster L. Hoffmann G. F. Kölker S. (2014). Emerging role of autophagy in pediatric neurodegenerative and neurometabolic diseases. *Pediatric Res.* 75 217–226. 10.1038/pr.2013.185 24165736

[B45] Eisele Y. S. Monteiro C. Fearns C. Encalada S. E. Wiseman R. L. Powers E. T. (2015). Targeting protein aggregation for the treatment of degenerative diseases. *Nat. Rev. Drug Discov.* 14 759–780. 10.1038/nrd4593 26338154 PMC4628595

[B46] Ellinger I. Pietschmann P. (2016). Endocytosis in health and disease—a thematic issue dedicated to Renate Fuchs. *Wien. Med. Wochenschr.* 166 193–195. 10.1007/s10354-016-0454-1 27165702

[B47] Emam S. E. Ando H. Lila A. S. A. Shimizu T. Okuhira K. Ishima Y. (2018). Liposome co-incubation with cancer cells secreted exosomes (extracellular vesicles) with different proteins expressions and different uptake pathways. *Sci. Rep.* 8:14493. 10.1038/s41598-018-32861-w PMC6160473 30262875

[B48] Escrevente C. Keller S. Altevogt P. Costa J. (2011). Interaction and uptake of exosomes by ovarian cancer cells. *BMC Cancer* 11:108. 10.1186/1471-2407-11-108 21439085 PMC3072949

[B49] Evans L. D. Wassmer T. Fraser G. Smith J. Perkinton M. Billinton A. (2018). Extracellular monomeric and aggregated tau efficiently enter human neurons through overlapping but distinct pathways. *Cell Rep.* 22 3612–3624. 10.1016/j.celrep.2018.03.021 29590627 PMC5896171

[B50] Fecto F. Yan J. Vemula S. P. Liu E. Yang Y. Chen W. (2011). SQSTM1 mutations in familial and sporadic amyotrophic lateral sclerosis. *Arch. Neurol.* 68:1440. 10.1001/archneurol.2011.250 22084127

[B51] Feng D. Zhao W. L. Ye Y. Y. Bai X. C. Liu R. Q. Chang L. F. (2010). Cellular internalization of exosomes occurs through phagocytosis. *Traffic* 11 675–687. 10.1111/j.1600-0854.2010.01041.x 20136776

[B52] Feng Y. He D. Yao Z. Klionsky D. J. (2014). The machinery of macroautophagy. *Cell Res.* 24 24–41. 10.1038/cr.2013.168 24366339 PMC3879710

[B53] Fiandaca M. S. Kapogiannis D. Mapstone M. Boxer A. Eitan E. Schwartz J. B. (2015). Identification of preclinical Alzheimer’s disease by a profile of pathogenic proteins in neurally derived blood exosomes: a case-control study. *Alzheimers Dement* 11 e600–e607. 10.1016/j.jalz.2014.06.008 PMC4329112 25130657

[B54] Fitzner D. Schnaars M. Van Rossum D. Krishnamoorthy G. Dibaj P. Bakhti M. (2011). Selective transfer of exosomes from oligodendrocytes to microglia by macropinocytosis. *J. Cell Sci.* 124 447–458. 10.1242/jcs.074088 21242314

[B55] Fred Dice J. (1990). Peptide sequences that target cytosolic proteins for lysosomal proteolysis. *Trends Biochem. Sci.* 15 305–309. 10.1016/0968-0004(90)90019-8 2204156

[B56] Fujiwara M. E. Zweifel N. (2018). Courtemanche, and T.D. Pollard, Latrunculin A accelerates actin filament depolymerization in addition to sequestering actin monomers. *Curr. Biol.* 28 e3183–e3192. 10.1016/j.cub.2018.07.082 PMC6179359 30270183

[B57] Fujiwara Y. Wada K. Kabuta T. (2017). Lysosomal degradation of intracellular nucleic acids—multiple autophagic pathways. *J. Biochem.* 161 145–154. 28039390 10.1093/jb/mvw085

[B58] Fürthauer M. Smythe E. (2014). Systems dynamics in endocytosis. *Traffic* 15 338–346. 10.1111/tra.12147 24405722

[B59] Gasser O. Hess C. Miot S. Deon C. Sanchez J.-C. Schifferli J. ÜA. (2003). Characterisation and properties of ectosomes released by human polymorphonuclear neutrophils. *Exp. Cell Res.* 285 243–257. 10.1016/s0014-4827(03)00055-7 12706119

[B60] Gaudin Y. Ruigrok R. H. Knossow M. Flamand A. (1993). Low-pH conformational changes of rabies virus glycoprotein and their role in membrane fusion. *J. Virol.* 67 1365–1372. 10.1128/jvi.67.3.1365-1372.1993 8437221 PMC237506

[B61] Genschmer K. R. Russell D. W. Lal C. Szul T. Bratcher P. E. Noerager B. D. (2019). Activated PMN exosomes: pathogenic entities causing matrix destruction and disease in the lung. *Cell* 176 e113–e126. 10.1016/j.cell.2018.12.002 PMC6368091 30633902

[B62] Gibbons G. S. Banks R. A. Kim B. Xu H. Changolkar L. Leight S. N. (2017). GFP-Mutant Human Tau transgenic mice develop tauopathy following CNS Injections of Alzheimer’s brain-derived pathological tau or synthetic mutant human tau fibrils. *J. Neurosci.* 37 11485–11494. 10.1523/jneurosci.2393-17.2017 28986461 PMC5700428

[B63] Goate M.-C. Chartier-Harlin M. Mullan J. Brown F. Crawford L. Fidani L. (1991). Segregation of a missense mutation in the amyloid precursor protein gene with familial Alzheimer’s disease. *Nature* 349 704–706. 1671712 10.1038/349704a0

[B64] Goedert M. (2015). Alzheimer’s and Parkinson’s diseases: the prion concept in relation to assembled Aβ, tau, and α-synuclein. *Science* 349:1255555. 10.1126/science.1255555 26250687

[B65] Gonda A. Kabagwira J. Senthil G. N. Wall N. R. (2019). Internalization of exosomes through receptor-mediated endocytosis. *Mol. Cancer Res.* 17 337–347. 10.1158/1541-7786.mcr-18-0891 30487244

[B66] Gribaudo S. Tixador P. Bousset L. Fenyi A. Lino P. Melki R. (2019). Propagation of α-Synuclein Strains within human reconstructed neuronal network. *Stem Cell Rep.* 12 230–244. 10.1016/j.stemcr.2018.12.007 30639210 PMC6372945

[B67] Grundke-Iqbal I. Iqbal K. Tung Y. C. Quinlan M. Wisniewski H. M. Binder L. I. (1986). Abnormal phosphorylation of the microtubule-associated protein tau (tau) in Alzheimer cytoskeletal pathology. *Proc. Natl. Acad. Sci. U. S. A.* 83 4913–4917. 10.1073/pnas.83.13.4913 3088567 PMC323854

[B68] Guix F. X. Corbett G. T. Cha D. J. Mustapic M. Liu W. Mengel D. (2018). Detection of aggregation-competent tau in neuron-derived extracellular vesicles. *Int. J. Mol. Sci.* 19:663. 10.3390/ijms19030663 29495441 PMC5877524

[B69] György B. Szabó T. G. Pásztói M. Pál Z. Misják P. Aradi B. (2011). Membrane vesicles, current state-of-the-art: emerging role of extracellular vesicles. *Cell. Mol. Life Sci.* 68 2667–2688. 10.1007/s00018-011-0689-3 21560073 PMC3142546

[B70] Ha K. D. Bidlingmaier S. M. Liu B. (2016). Macropinocytosis exploitation by cancers and cancer therapeutics. *Front. Physiol.* 7:381. 10.3389/fphys.2016.00381 27672367 PMC5018483

[B71] Haass C. Selkoe D. J. (1993). Cellular processing of β-amyloid precursor protein and the genesis of amyloid β-peptide. *Cell* 75 1039–1042. 10.1016/0092-8674(93)90312-e 8261505

[B72] Hallinan G. I. Vargas-Caballero M. West J. Deinhardt K. (2019). Tau misfolding efficiently propagates between individual intact hippocampal neurons. *J. Neurosci.* 39 9623–9632. 10.1523/jneurosci.1590-19.2019 31658988 PMC6880463

[B73] Hara T. Nakamura K. Matsui M. Yamamoto A. Nakahara Y. Suzuki-Migishima R. (2006). Suppression of basal autophagy in neural cells causes neurodegenerative disease in mice. *Nature* 441 885–889. 10.1038/nature04724 16625204

[B74] Harding C. Heuser J. Stahl P. (1983). Receptor-mediated endocytosis of transferrin and recycling of the transferrin receptor in rat reticulocytes. *J. Cell Biol.* 97 329–339. 10.1083/jcb.97.2.329 6309857 PMC2112509

[B75] Harischandra D. S. Rokad D. Neal M. L. Ghaisas S. Manne S. Sarkar S. (2019). Manganese promotes the aggregation and prion-like cell-to-cell exosomal transmission of α-synuclein. *Sci. Signal.* 12:eaau4543. 10.1126/scisignal.aau4543 30862700 PMC6435331

[B76] Harper J. D. Lansbury P. T. Jr. (1997). Models of amyloid seeding in Alzheimer’s disease and scrapie: mechanistic truths and physiological consequences of the time-dependent solubility of amyloid proteins. *Annu. Rev. Biochem.* 66 385–407. 10.1146/annurev.biochem.66.1.385 9242912

[B77] Hassiotis S. Manavis J. Blumbergs P. C. Hattersley K. J. Carosi J. M. Kamei M. (2018). Lysosomal LAMP1 immunoreactivity exists in both diffuse and neuritic amyloid plaques in the human hippocampus. *Eur. J. Neurosci.* 47 1043–1053. 10.1111/ejn.13913 29570886

[B78] Hazan-Halevy I. Rosenblum D. Weinstein S. Bairey O. Raanani P. Peer D. (2015). Cell-specific uptake of mantle cell lymphoma-derived exosomes by malignant and non-malignant B-lymphocytes. *Cancer Lett.* 364 59–69. 10.1016/j.canlet.2015.04.026 25933830 PMC4490183

[B79] He C. Klionsky D. J. (2009). Regulation mechanisms and signaling pathways of autophagy. *Annu. Rev. Genet.* 43 67–93. 10.1146/annurev-genet-102808-114910 19653858 PMC2831538

[B80] Henderson M. X. Cornblath E. J. Darwich A. Zhang B. Brown H. Gathagan R. J. (2019). Spread of α-synuclein pathology through the brain connectome is modulated by selective vulnerability and predicted by network analysis. *Nat. Neurosci.* 22 1248–1257. 10.1038/s41593-019-0457-5 31346295 PMC6662627

[B81] Heusermann W. Hean J. Trojer D. Steib E. Von Bueren S. Graff-Meyer A. (2016). Exosomes surf on filopodia to enter cells at endocytic hot spots, traffic within endosomes, and are targeted to the ER. *J. Cell Biol.* 213 173–184. 10.1083/jcb.201506084 27114500 PMC5084269

[B82] Hill E. Van Der Kaay J. Downes C. P. Smythe E. (2001). The role of dynamin and its binding partners in coated pit invagination and scission. *J. Cell Biol.* 152 309–324. 10.1083/jcb.152.2.309 11266448 PMC2199618

[B83] Hoffmann A.-C. Minakaki G. Menges S. Salvi R. Savitskiy S. Kazman A. (2019). Extracellular aggregated alpha synuclein primarily triggers lysosomal dysfunction in neural cells prevented by trehalose. *Sci. Rep.* 9:544. 10.1038/s41598-018-35811-8 PMC6345801 30679445

[B84] Hoffmann P. R. Decathelineau A. M. Ogden C. A. Leverrier Y. Bratton D. L. Daleke D. L. (2001). Phosphatidylserine (PS) induces PS receptor–mediated macropinocytosis and promotes clearance of apoptotic cells. *J. Cell Biol.* 155 649–660. 10.1083/jcb.200108080 11706053 PMC2198875

[B85] Holder B. Jones T. Sancho Shimizu V. Rice T. F. Donaldson B. Bouqueau M. (2016). Macrophage exosomes induce placental inflammatory cytokines: a novel mode of maternal-placental messaging. *Traffic* 17 168–178. 10.1111/tra.12352 26602702 PMC4738478

[B86] Horibe S. Tanahashi T. Kawauchi S. Murakami Y. Rikitake Y. (2018). Mechanism of recipient cell-dependent differences in exosome uptake. *BMC Cancer* 18:47. 10.1186/s12885-017-3958-1 29306323 PMC5756423

[B87] Hu X. Crick S. L. Bu G. Frieden C. Pappu R. V. Lee J. M. (2009). Amyloid seeds formed by cellular uptake, concentration, and aggregation of the amyloid-beta peptide. *Proc. Natl. Acad. Sci. U. S. A.* 106 20324–20329. 10.1073/pnas.0911281106 19910533 PMC2787156

[B88] Hussein H. A. M. Walker L. R. Abdel-Raouf U. M. Desouky S. A. Montasser A. K. M. Akula S. M. (2015). Beyond RGD: virus interactions with integrins. *Arch. Virol.* 160 2669–2681. 10.1007/s00705-015-2579-8 26321473 PMC7086847

[B89] Igami K. Uchiumi T. Ueda S. Kamioka K. Setoyama D. Gotoh K. (2020). Characterization and function of medium and large extracellular vesicles from plasma and urine by surface antigens and Annexin V. *PeerJ Anal. Chem.* 2:e4 10.7717/peerj-achem.4

[B90] Ilahibaks N. F. Lei Z. Mol E. A. Deshantri A. K. Jiang L. Schiffelers R. M. (2019). Biofabrication of cell-derived nanovesicles: a potential alternative to extracellular vesicles for regenerative medicine. *Cells* 8:1509. 10.3390/cells8121509 31775322 PMC6952804

[B91] Joseph J. G. Liu A. P. (2020). Mechanical regulation of endocytosis: new insights and recent advances. *Adv. Biosyst.* 4:1900278. 10.1002/adbi.201900278 33179871

[B92] Joshi B. S. De Beer M. A. Giepmans B. N. Zuhorn I. S. (2020). Endocytosis of extracellular vesicles and release of their cargo from endosomes. *ACS Nano* 4 4444–4455. 10.1021/acsnano.9b10033 32282185 PMC7199215

[B93] Kaksonen M. Roux A. (2018). Mechanisms of clathrin-mediated endocytosis. *Nat. Rev. Mol. Cell Biol.* 19 313–326. 29410531 10.1038/nrm.2017.132

[B94] Karpowicz R. J. Jr. Haney C. M. Mihaila T. S. Sandler R. M. Petersson E. J. Lee V. M. (2017). Selective imaging of internalized proteopathic α-synuclein seeds in primary neurons reveals mechanistic insight into transmission of synucleinopathies. *J. Biol. Chem.* 292 13482–13497. 10.1074/jbc.m117.780296 28611062 PMC5555207

[B95] Kaufman S. K. Del Tredici K. Thomas T. L. Braak H. Diamond M. I. (2018). Tau seeding activity begins in the transentorhinal/entorhinal regions and anticipates phospho-tau pathology in Alzheimer’s disease and PART. *Acta Neuropathol.* 136 57–67. 10.1007/s00401-018-1855-6 29752551 PMC6015098

[B96] Kerr M. C. Teasdale R. D. (2009). Defining macropinocytosis. *Traffic* 10 364–371. 10.1111/j.1600-0854.2009.00878.x 19192253

[B97] Kettler K. Giannakou C. De Jong W. H. Hendriks A. J. Krystek P. (2016). Uptake of silver nanoparticles by monocytic THP-1 cells depends on particle size and presence of serum proteins. *J. Nanopart. Res.* 18:286. 10.1007/s11051-016-3595-7 PMC5034003 27774037

[B98] Kim M. Sandford E. Gatica D. Qiu Y. Liu X. Zheng Y. (2016). Mutation in ATG5 reduces autophagy and leads to ataxia with developmental delay. *eLife* 5:e12245. 10.7554/eLife.12245 PMC4786408 26812546

[B99] Kirchhausen T. (2000). Clathrin. *Annu. Rev. Biochem.* 69 699–727. 10966473 10.1146/annurev.biochem.69.1.699

[B100] Koivusalo M. Welch C. Hayashi H. Scott C. C. Kim M. Alexander T. (2010). Amiloride inhibits macropinocytosis by lowering submembranous pH and preventing Rac1 and Cdc42 signaling. *J. Cell Biol.* 188 547–563. 10.1083/jcb.200908086 20156964 PMC2828922

[B101] Kooijmans S. A. A. Schiffelers R. M. Zarovni N. Vago R. (2016). Modulation of tissue tropism and biological activity of exosomes and other extracellular vesicles: new nanotools for cancer treatment. *Pharmacol. Res.* 111 487–500. 10.1016/j.phrs.2016.07.006 27394168

[B102] Koumangoye R. B. Sakwe A. M. Goodwin J. S. Patel T. Ochieng J. (2011). Detachment of breast tumor cells induces rapid secretion of exosomes which subsequently mediate cellular adhesion and spreading. *PLoS One* 6:e24234. 10.1371/journal.pone.0024234 21915303 PMC3167827

[B103] Kovács G. G. Gelpi E. Ströbel T. Ricken G. Nyengaard J. R. Bernheimer H. (2007). Involvement of the endosomal-lysosomal system correlates with regional pathology in creutzfeldt-jakob disease. *J. Neuropathol. Exp. Neurol.* 66 628–636. 10.1097/nen.0b013e318093ecc7 17620988

[B104] Kruth H. S. Jones N. L. Huang W. Zhao B. Ishii I. Chang J. (2005). Macropinocytosis is the endocytic pathway that mediates macrophage foam cell formation with native low density lipoprotein. *J. Biol. Chem.* 280 2352–2360. 10.1074/jbc.m407167200 15533943

[B105] Kuhn D. A. Vanhecke D. Michen B. Blank F. Gehr P. Petri-Fink A. (2014). Different endocytotic uptake mechanisms for nanoparticles in epithelial cells and macrophages. *Beilstein J. Nanotechnol.* 5 1625–1636. 10.3762/bjnano.5.174 25383275 PMC4222452

[B106] Kulkarni R. Bajaj M. Ghode S. Jalnapurkar S. Limaye L. Kale V. P. (2018). Intercellular transfer of microvesicles from young mesenchymal stromal cells rejuvenates aged murine hematopoietic stem cells. *Stem Cell.* 36 420–433. 10.1002/stem.2756 29230885

[B107] Kwok C. T. Morris A. de Belleroche J. S. (2014). Sequestosome-1 (SQSTM1) sequence variants in ALS cases in the UK: prevalence and coexistence of SQSTM1 mutations in ALS kindred with PDB. *Eur. J. Hum. Genet.* 22 492–496. 10.1038/ejhg.2013.184 23942205 PMC3953910

[B108] Lafon M. (2005). Rabies virus receptors. *J. Neurovirol.* 11 82–87. 10.1080/13550280590900427 15804965

[B109] Lamark T. Johansen T. (2012). Aggrephagy: selective disposal of protein aggregates by macroautophagy. *Int. J. Cell Biol.* 2012:736905. 10.1155/2012/736905 PMC3320095 22518139

[B110] Lamaze C. Fujimoto L. M. Yin H. L. Schmid S. L. (1997). The actin cytoskeleton is required for receptor-mediated endocytosis in mammalian cells. *J. Biol. Chem.* 272 20332–20335. 10.1074/jbc.272.33.20332 9252336

[B111] Lawrence R. E. Zoncu R. (2019). The lysosome as a cellular centre for signalling, metabolism and quality control. *Nat. Cell Biol.* 21 133–142. 10.1038/s41556-018-0244-7 30602725

[B112] Lee H.-J. Khoshaghideh F. Patel S. Lee S.-J. (2004). Clearance of α-Synuclein oligomeric intermediates via the lysosomal degradation pathway. *J. Neurosci.* 24 1888–1896. 10.1523/jneurosci.3809-03.2004 14985429 PMC6730405

[B113] Lee H.-J. Yoon Y.-S. Lee S.-J. (2018). Mechanism of neuroprotection by trehalose: controversy surrounding autophagy induction. *Cell Death Dis.* 9:712. 10.1038/s41419-018-0749-9 PMC6003909 29907758

[B114] Lentz T. Burrage T. Smith A. Crick J. Tignor G. (1982). Is the acetylcholine receptor a rabies virus receptor? *Science* 215 182–184. 10.1126/science.7053569 7053569

[B115] Li W.-W. Li J. Bao J.-K. (2012). Microautophagy: lesser-known self-eating. *Cell. Mol. Life Sci.* 69 1125–1136. 10.1007/s00018-011-0865-5 22080117 PMC11114512

[B116] Li Y. Xu M. Ding X. Yan C. Song Z. Chen L. (2016). Protein kinase C controls lysosome biogenesis independently of mTORC1. *Nat. Cell Biol.* 18 1065–1077. 10.1038/ncb3407 27617930

[B117] Lim J. Yue Z. (2015). Neuronal aggregates: formation, clearance, and spreading. *Dev. Cell* 32 491–501. 10.1016/j.devcel.2015.02.002 25710535 PMC4376477

[B118] Lim J. P. Gleeson P. A. (2011). Macropinocytosis: an endocytic pathway for internalising large gulps. *Immunol. Cell Biol.* 89 836–843. 10.1038/icb.2011.20 21423264

[B119] Lin H.-P. Singla B. Ghoshal P. Faulkner J. L. Cherian-Shaw M. O’Connor P. M. (2018). De Chantemele, and G. Csányi, Identification of novel macropinocytosis inhibitors using a rational screen of Food and Drug Administration-approved drugs. *Br. J. Pharmacol.* 175 3640–3655. 10.1111/bph.14429 29953580 PMC6109223

[B120] Lott I. T. Head E. (2019). Dementia in Down syndrome: unique insights for Alzheimer disease research. *Nat. Rev. Neurol.* 15 135–147. 10.1038/s41582-018-0132-6 30733618 PMC8061428

[B121] Luan X. Sansanaphongpricha K. Myers I. Chen H. Yuan H. Sun D. (2017). Engineering exosomes as refined biological nanoplatforms for drug delivery. *Acta Pharmacol. Sinica* 38 754–763. 10.1038/aps.2017.12 28392567 PMC5520184

[B122] Lucin K. M. O’Brien C. E. Bieri G. Czirr E. Mosher K. I. Abbey R. J. (2013). Microglial Beclin 1 regulates retromer trafficking and phagocytosis and is impaired in Alzheimer’s Disease. *Neuron* 79 873–886. 10.1016/j.neuron.2013.06.046 24012002 PMC3779465

[B123] Lwin A. Orvisky E. Goker-Alpan O. LaMarca M. E. Sidransky E. (2004). Glucocerebrosidase mutations in subjects with parkinsonism. *Mol. Genet. Metab.* 81 70–73. 10.1016/j.ymgme.2003.11.004 14728994

[B124] Macia E. Ehrlich M. Massol R. Boucrot E. Brunner C. Kirchhausen T. (2006). Dynasore, a cell-permeable inhibitor of dynamin. *Dev. Cell* 10 839–850. 10.1016/j.devcel.2006.04.002 16740485

[B125] Malik B. R. Maddison D. C. Smith G. A. Peters O. M. (2019). Autophagic and endo-lysosomal dysfunction in neurodegenerative disease. *Mol. Brain* 12:100. 10.1186/s13041-019-0504-x PMC6884906 31783880

[B126] Martinez-Vicente M. Talloczy Z. Kaushik S. Massey A. C. Mazzulli J. Mosharov E. V. (2008). Dopamine-modified α-synuclein blocks chaperone-mediated autophagy. *J. Clin. Invest.* 182 777–788. 10.1172/JCI32806 PMC2157565 18172548

[B127] Martinez-Vicente M. Talloczy Z. Wong E. Tang G. Koga H. Kaushik S. (2010). Cargo recognition failure is responsible for inefficient autophagy in Huntington’s disease. *Nat. Neurosci.* 13 567–576. 10.1038/nn.2528 20383138 PMC2860687

[B128] Massey A. C. Kaushik S. Sovak G. Kiffin R. Cuervo A. M. (2006). Consequences of the selective blockage of chaperone-mediated autophagy. *Proc. Natl. Acad. Sci. U. S. A.* 103 5805–5810. 10.1073/pnas.0507436103 16585521 PMC1458654

[B129] Masters C. L. Multhaup G. Simms G. Pottgiesser J. Martins R. N. Beyreuther K. (1985). Neuronal origin of a cerebral amyloid: neurofibrillary tangles of Alzheimer’s disease contain the same protein as the amyloid of plaque cores and blood vessels. *EMBO J.* 4 2757–2763. 10.1002/j.1460-2075.1985.tb04000.x 4065091 PMC554575

[B130] Mathieu M. Martin-Jaular L. Lavieu G. Thery C. (2019). Specificities of secretion and uptake of exosomes and other extracellular vesicles for cell-to-cell communication. *Nat. Cell Biol.* 21 9–17. 10.1038/s41556-018-0250-9 30602770

[B131] Matsudaira T. Mukai K. Noguchi T. Hasegawa J. Hatta T. Iemura S.-I. (2017). Endosomal phosphatidylserine is critical for the YAP signalling pathway in proliferating cells. *Nat. Commun.* 8:1246. 10.1038/s41467-017-01255-3 PMC5665887 29093443

[B132] Mauthe M. Orhon I. Rocchi C. Zhou X. Luhr M. Hijlkema K. J. (2018). Chloroquine inhibits autophagic flux by decreasing autophagosome-lysosome fusion. *Autophagy* 14 1435–1455. 10.1080/15548627.2018.1474314 29940786 PMC6103682

[B133] Mauvezin C. Neufeld T. P. (2015). Bafilomycin A1 disrupts autophagic flux by inhibiting both V-ATPase-dependent acidification and Ca-P60A/SERCA-dependent autophagosome-lysosome fusion. *Autophagy* 11 1437–1438. 10.1080/15548627.2015.1066957 26156798 PMC4590655

[B134] Mazulli R. J. Xu Y.-H. Sun Y. Knight A. L. McLean P. J. Caldwell G. A. (2011). Gaucher Disease Glucocerebrosidase and α-Synuclein form a bidirectional pathogenic loop in synucleinopathies. *Cell* 146 37–52. 10.1016/j.cell.2011.06.001 21700325 PMC3132082

[B135] McGeachie A. B. Odell L. R. Quan A. Daniel J. A. Chau N. Hill T. A. (2013). Pyrimidyn compounds: dual-action small molecule pyrimidine-based dynamin inhibitors. *ACS Chem. Biol.* 8 1507–1518. 10.1021/cb400137p 23642287

[B136] Mellman I. (1996). Endocytosis and molecular sorting. *Annu. Rev. Cell Dev. Biol.* 12 575–625. 10.1146/annurev.cellbio.12.1.575 8970738

[B137] Menzies F. M. Fleming A. Caricasole A. Bento C. F. Andrews S. P. Ashkenazi A. (2017). Autophagy and Neurodegeneration: pathogenic mechanisms and therapeutic opportunities. *Neuron* 93 1015–1034. 10.1016/j.neuron.2017.01.022 28279350

[B138] Mercer J. Helenius A. (2009). Virus entry by macropinocytosis. *Nat. Cell Biol.* 11 510–520. 10.1038/ncb0509-510 19404330

[B139] Mercer J. Schelhaas M. Helenius A. (2010). Virus entry by endocytosis. *Annu. Rev. Biochem.* 79 803–833. 10.1146/annurev-biochem-060208-104626 20196649

[B140] Metaxakis A. Ploumi C. Tavernarakis N. (2018). Autophagy in age-associated neurodegeneration. *Cells* 7:37. 10.3390/cells7050037 29734735 PMC5981261

[B141] Miaczynska M. Pelkmans L. Zerial M. (2004). Not just a sink: endosomes in control of signal transduction. *Curr. Opin. Cell Biol.* 16 400–406. 10.1016/j.ceb.2004.06.005 15261672

[B142] Miaczynska M. Stenmark H. (2008). Mechanisms and functions of endocytosis: figure 1. *J. Cell Biol.* 180 7–11. 10.1083/jcb.200711073 18195098 PMC2213624

[B143] Mijaljica D. Prescott M. Devenish R. J. (2011). Microautophagy in mammalian cells: revisiting a 40-year-old conundrum. *Autophagy* 7 673–682. 10.4161/auto.7.7.14733 21646866

[B144] Mindell J. A. (2012). Lysosomal acidification mechanisms. *Annu. Rev. Physiol.* 74 69–86. 10.1146/annurev-physiol-012110-142317 22335796

[B145] Mizushima N. (2018). A brief history of autophagy from cell biology to physiology and disease. *Nat. Cell Biol.* 20 521–527. 10.1038/s41556-018-0092-5 29686264

[B146] Mizushima N. Komatsu M. (2011). Autophagy: renovation of cells and tissues. *Cell* 147 728–741. 10.1016/j.cell.2011.10.026 22078875

[B147] Mizushima N. Levine B. Cuervo A. M. Klionsky D. J. (2008). Autophagy fights disease through cellular self-digestion. *Nature* 451 1069–1075. 10.1038/nature06639 18305538 PMC2670399

[B148] Montecalvo A. T. Larregina W.J. Shufesky Stolz D. Beer Sullivan M. L. G. Karlsson J. M. Baty C. J. (2012). Mechanism of transfer of functional microRNAs between mouse dendritic cells via exosomes. *Blood* 119 756–766. 10.1182/blood-2011-02-338004 22031862 PMC3265200

[B149] Morelli A. E. Larregina A. T. Shufesky W. J. Sullivan M. L. Stolz D. B. Papworth G. D. (2004). Endocytosis, intracellular sorting, and processing of exosomes by dendritic cells. *Blood* 104 3257–3266. 10.1182/blood-2004-03-0824 15284116

[B150] Mulcahy L. A. Pink R. C. Carter D. R. F. (2014). Routes and mechanisms of extracellular vesicle uptake. *J. Extracell. Vesicles* 3:24641. 10.3402/jev.v3.24641 25143819 PMC4122821

[B151] Nakamura S. Yoshimori T. (2017). New insights into autophagosome–lysosome fusion. *J. Cell Sci.* 130 1209–1216. 10.1242/jcs.196352 28302910

[B152] Nakase I. Kobayashi N. B. Takatani-Nakase T. Yoshida T. (2015). Active macropinocytosis induction by stimulation of epidermal growth factor receptor and oncogenic Ras expression potentiates cellular uptake efficacy of exosomes. *Sci. Rep.* 5:10300. 10.1038/srep10300 PMC4453128 26036864

[B153] Nakase I. Noguchi K. Fujii I. Futaki S. (2016). Vectorization of biomacromolecules into cells using extracellular vesicles with enhanced internalization induced by macropinocytosis. *Sci. Rep.* 6:34937. 10.1038/srep34937 PMC5066177 27748399

[B154] Nalbandian A. Llewellyn K. J. Nguyen C. Yazdi P. G. Kimonis V. E. (2015). Rapamycin and Chloroquine: the *In Vitro* and In Vivo effects of autophagy-modifying drugs show promising results in valosin containing protein multisystem proteinopathy. *PLoS One* 10:e0122888. 10.1371/journal.pone.0122888 25884947 PMC4401571

[B155] Nanbo A. Kawanishi E. Yoshida R. Yoshiyama H. (2013). Exosomes derived from epstein-barr virus-infected cells are internalized via caveola-dependent endocytosis and promote phenotypic modulation in target cells. *J. Virol.* 87 10334–10347. 10.1128/jvi.01310-13 23864627 PMC3753980

[B156] Narasimhan S. Guo J. L. Changolkar L. Stieber A. McBride J. D. Silva L. V. (2017). Pathological Tau strains from human brains recapitulate the diversity of tauopathies in nontransgenic mouse brain. *J. Neurosci.* 37 11406–11423. 10.1523/jneurosci.1230-17.2017 29054878 PMC5700423

[B157] Neumann M. Sampathu D. M. Kwong L. K. Truax A. C. Micsenyi M. C. Chou T. T. (2006). Ubiquitinated TDP-43 in frontotemporal lobar degeneration and amyotrophic lateral sclerosis. *Science* 314 130–133. 17023659 10.1126/science.1134108

[B158] Nilsson P. Loganathan K. Sekiguchi M. Matsuba Y. Hui K. Tsubuki S. (2013). Aβ secretion and plaque formation depend on autophagy. *Cell Rep.* 5 61–69. 10.1016/j.celrep.2013.08.042 24095740

[B159] Nixon R. A. Wegiel J. Kumar A. Yu W. H. Peterhoff C. Cataldo A. (2005). Extensive involvement of autophagy in Alzheimer disease: an immuno-electron microscopy study. *J. Neuropathol. Exp. Neurol.* 64 113–122. 10.1093/jnen/64.2.113 15751225

[B160] Nolte-‘T Hoen E. N. M. Buschow S. I. Anderton S. M. Stoorvogel W. Wauben M. H. M. (2009). Activated T cells recruit exosomes secreted by dendritic cells via LFA-1. *Blood* 113 1977–1981. 10.1182/blood-2008-08-174094 19064723

[B161] O’Brien K. Breyne K. Ughetto S. Laurent L. C. Breakefield X. O. (2020). RNA delivery by extracellular vesicles in mammalian cells and its applications. *Nat. Rev. Mol. Cell Biol.* 21 585–606. 10.1038/s41580-020-0251-y 32457507 PMC7249041

[B162] Ochaba J. Lukacsovich T. Csikos G. Zheng S. Margulis J. Salazar L. (2014). Potential function for the Huntingtin protein as a scaffold for selective autophagy. *Proc. Natl. Acad. Sci. U. S. A.* 111 16889–16894. 10.1073/pnas.1420103111 25385587 PMC4250109

[B163] Odagiri S. Tanji K. Mori F. Kakita A. Takahashi H. Wakabayashi K. (2012). Autophagic adapter protein NBR1 is localized in Lewy bodies and glial cytoplasmic inclusions and is involved in aggregate formation in α-synucleinopathy. *Acta Neuropathol.* 124 173–186. 10.1007/s00401-012-0975-7 22484440

[B164] Ogese M. O. Jenkins R. E. Adair K. Tailor A. Meng X. Faulkner L. (2019). Exosomal transport of hepatocyte−derived drug−modified proteins to the immune system. *Hepatology* 70 1732–1749. 10.1002/hep.30701 31070244 PMC6899733

[B165] Oh P. McIntosh D. P. Schnitzer J. E. (1998). Dynamin at the neck of caveolae mediates their budding to form transport vesicles by GTP-driven fission from the plasma membrane of endothelium. *J. Cell Biol.* 141 101–114. 10.1083/jcb.141.1.101 9531551 PMC2132716

[B166] Orenstein S. J. Cuervo A. M. (2010). Chaperone-mediated autophagy: molecular mechanisms and physiological relevance. *Semin. Cell Dev. Biol.* 21 719–726. 10.1016/j.semcdb.2010.02.005 20176123 PMC2914824

[B167] Osawa T. Mizuno Y. Fujita Y. Takatama M. Nakazato Y. Okamoto K. (2011). Optineurin in neurodegenerative diseases. *Neuropathology* 31 569–574. 10.1111/j.1440-1789.2011.01199.x 21284751

[B168] Osellame L. D. Rahim A. A. Hargreaves I. P. Gegg M. E. Richard-Londt A. Brandner S. (2013). Mitochondria and quality control defects in a mouse model of gaucher disease—links to Parkinson’s Disease. *Cell Metab.* 17 941–953. 10.1016/j.cmet.2013.04.014 23707074 PMC3678026

[B169] Øverbye M. F. (2007). Brinchmann, and P.O. seglen, proteomic analysis of membrane-associated proteins from rat liver autophagosomes. *Autophagy* 3 300–322. 10.4161/auto.3910 17377489

[B170] Pan B. T. (1985). Electron microscopic evidence for externalization of the transferrin receptor in vesicular form in sheep reticulocytes. *J. Cell Biol.* 101 942–948. 10.1083/jcb.101.3.942 2993317 PMC2113705

[B171] Pan B.-T. Johnstone R. M. (1983). Fate of the transferrin receptor during maturation of sheep reticulocytes *in Vitro*: selective externalization of the receptor. *Cell* 33 967–978. 10.1016/0092-8674(83)90040-5 6307529

[B172] Pan K. M. Baldwin M. Nguyen J. Gasset M. Serban A. Groth D. (1993). Conversion of alpha-helices into beta-sheets features in the formation of the scrapie prion proteins. *Proc. Natl. Acad. Sci. U. S. A.* 90 10962–10966. 10.1073/pnas.90.23.10962 7902575 PMC47901

[B173] Pan Q. Ramakrishnaiah V. Henry S. Fouraschen S. De Ruiter P. E. Kwekkeboom J. (2012). Hepatic cell-to-cell transmission of small silencing RNA can extend the therapeutic reach of RNA interference (RNAi). *Gut* 61 1330–1339. 10.1136/gutjnl-2011-300449 22198713

[B174] Pandey U. B. Nie Z. Batlevi Y. McCray B. A. Ritson G. P. Nedelsky N. B. (2007). HDAC6 rescues neurodegeneration and provides an essential link between autophagy and the UPS. *Nature* 447 859–863. 17568747 10.1038/nature05853

[B175] Pankiv S. Clausen T. H. Lamark T. Brech A. Bruun J.-A. Outzen H. (2007). p62/SQSTM1 Binds Directly to Atg8/LC3 to facilitate degradation of ubiquitinated protein aggregates by autophagy. *J. Biol. Chem.* 282 24131–24145. 10.1074/jbc.m702824200 17580304

[B176] Parolini I. Federici C. Raggi C. Lugini L. Palleschi S. Milito A. De, et al. (2009). Microenvironmental pH Is a key factor for exosome traffic in tumor cells. *J. Biol. Chem.* 284 34211–34222. 10.1074/jbc.m109.041152 19801663 PMC2797191

[B177] Parton R. G. Collins B. M. (2016). Unraveling the architecture of caveolae. *Proc. Natl. Acad. Sci. U. S. A.* 113 14170–14172. 10.1073/pnas.1617954113 27911845 PMC5167180

[B178] Pasternak S. H. Callahan J. W. Mahuran D. J. (2004). The role of the endosomal/lysosomal system in amyloid-beta production and the pathophysiology of Alzheimer’s disease: reexamining the spatial paradox from a lysosomal perspective. *J. Alzheimers Dis.* 6 53–65. 10.3233/jad-2004-6107 15004328

[B179] Pedrioli G. Barberis M. Molinari M. Morone D. Papin S. Paganetti P. (2020). Extracellular vesicles hijack the autophagic pathway to induce tau accumulation in endolysosomes. *bioRxiv [preprint]* 10.1101/2020.05.27.118323

[B180] Pfrieger F. W. Vitale N. (2018). Cholesterol and the journey of extracellular vesicles. *J. Lipid Res.* 59 2255–2261. 10.1194/jlr.r084210 29678958 PMC6277151

[B181] Pickford F. Masliah E. Britschgi M. Lucin K. Narasimhan R. Jaeger P. A. (2008). The autophagy-related protein beclin 1 shows reduced expression in early Alzheimer disease and regulates amyloid β accumulation in mice. *J. Clin. Invest.* 118 2190–2199. 18497889 10.1172/JCI33585 PMC2391284

[B182] Piras A. Collin L. Grüninger F. Graff C. Rönnbäck A. (2016). Autophagic and lysosomal defects in human tauopathies: analysis of post-mortem brain from patients with familial Alzheimer disease, corticobasal degeneration and progressive supranuclear palsy. *Acta Neuropathol. Commun.* 4:22. 10.1186/s40478-016-0292-9 PMC4774096 26936765

[B183] Polanco J. C. Li C. Durisic N. Sullivan R. Götz J. (2018). Exosomes taken up by neurons hijack the endosomal pathway to spread to interconnected neurons. *Acta Neuropathol. Commun.* 6:10. 10.1186/s40478-018-0514-4 PMC5815204 29448966

[B184] Polymeropoulos M. H. Lavedan C. Leroy E. Ide S. E. Dehejia A. Dutra A. (1997). Mutation in the a-synuclein gene identified in families with Parkinson’s disease. *Science* 276 2045–2047. 10.1126/science.276.5321.2045 9197268

[B185] Poulson B. G. Szczepski K. Lachowicz J. I. Jaremko L. Emwas A.-H. Jaremko M. (2020). Aggregation of biologically important peptides and proteins: inhibition or acceleration depending on protein and metal ion concentrations. *RSC Adv.* 10 215–227. 10.1039/c9ra09350h PMC9047971 35492549

[B186] Prada I. Meldolesi J. (2016). Binding and fusion of extracellular vesicles to the plasma membrane of their cell targets. *Int. J. Mol. Sci.* 17:1296. 10.3390/ijms17081296 27517914 PMC5000693

[B187] Ramesh Babu J. Lamar Seibenhener M. Peng J. Strom A. L. Kemppainen R. Cox N. (2008). Genetic inactivation of p62 leads to accumulation of hyperphosphorylated tau and neurodegeneration. *J. Neurochem.* 106 107–120. 10.1111/j.1471-4159.2008.05340.x 18346206

[B188] Raposo G. Nijman H. W. Stoorvogel W. Liejendekker R. Harding C. V. Melief C. J. (1996). B lymphocytes secrete antigen-presenting vesicles. *J. Exp. Med.* 183 1161–1172. 10.1084/jem.183.3.1161 8642258 PMC2192324

[B189] Raposo G. Stoorvogel W. (2013). Extracellular vesicles: exosomes, microvesicles, and friends. *J. Cell Biol.* 200 373–383. 10.1083/jcb.201211138 23420871 PMC3575529

[B190] Rappa G. Santos M. F. Green T. M. Karbanová J. Hassler J. Bai Y. (2017). Nuclear transport of cancer extracellular vesicle-derived biomaterials through nuclear envelope invagination-associated late endosomes. *Oncotarget* 8 14443–14461. 10.18632/oncotarget.14804 28129640 PMC5362417

[B191] Ravikumar B. Duden R. Rubinsztein D. C. (2002). Aggregate-prone proteins with polyglutamine and polyalanine expansions are degraded by autophagy. *Hum. Mol. Genet.* 11 1107–1117. 10.1093/hmg/11.9.1107 11978769

[B192] Ravikumar B. Vacher C. Berger Z. Davies J. E. Luo S. Oroz L. G. (2004). Inhibition of mTOR induces autophagy and reduces toxicity of polyglutamine expansions in fly and mouse models of Huntington disease. *Nat. Genet.* 36 585–595. 10.1038/ng1362 15146184

[B193] Richards D. M. Endres R. G. (2014). The mechanism of phagocytosis: two stages of engulfment. *Biophys. J.* 107 1542–1553. 10.1016/j.bpj.2014.07.070 25296306 PMC4190621

[B194] Ridder K. Keller S. Dams M. Rupp A.-K. Schlaudraff J. Del Turco D. (2014). Extracellular vesicle-mediated transfer of genetic information between the hematopoietic system and the brain in response to inflammation. *PLoS Biol.* 12:e1001874. 10.1371/journal.pbio.1001874 24893313 PMC4043485

[B195] Ridder K. Sevko A. Heide J. Dams M. Rupp A.-K. Macas J. (2015). Extracellular vesicle-mediated transfer of functional RNA in the tumor microenvironment. *OncoImmunology* 4:e1008371. 10.1080/2162402x.2015.1008371 26155418 PMC4485784

[B196] Rosales C. Uribe-Querol E. (2017). Phagocytosis: a fundamental process in immunity. *BioMed Res. Int.* 2017:9042851. 10.1155/2017/9042851 PMC5485277 28691037

[B197] Ross C. A. Poirier M. A. (2005). Opinion: what is the role of protein aggregation in neurodegeneration? *Nat. Rev. Mol. Cell Biol.* 6 891–898. 10.1038/nrm1742 16167052

[B198] Rubartelli A. Poggi A. Zocchi M. R. (1997). The selective engulfment of apoptotic bodies by dendritic cells is mediated by the αvβ3 integrin and requires intracellular and extracellular calcium. *Eur. J. Immunol.* 27 1893–1900. 10.1002/eji.1830270812 9295024

[B199] Sahu R. Kaushik S. Clement C. C. Cannizzo E. S. Scharf B. Follenzi A. (2011). Microautophagy of cytosolic proteins by late endosomes. *Dev. Cell* 20 131–139. 10.1016/j.devcel.2010.12.003 21238931 PMC3025279

[B200] Sallusto F. (1995). Dendritic cells use macropinocytosis and the mannose receptor to concentrate macromolecules in the major histocompatibility complex class II compartment: downregulation by cytokines and bacterial products. *J. Exp. Med.* 182 389–400. 10.1084/jem.182.2.389 7629501 PMC2192110

[B201] Sarkar S. Davies J. E. Huang Z. Tunnacliffe A. Rubinsztein D. C. (2007). Trehalose, a Novel mTOR-independent autophagy enhancer, accelerates the clearance of mutant huntingtin and α-synuclein. *J. Biol. Chem.* 282 5641–5652. 10.1074/jbc.m609532200 17182613

[B202] Savina A. Amigorena S. (2007). Phagocytosis and antigen presentation in dendritic cells. *Immunol. Rev.* 219 143–156. 10.1111/j.1600-065x.2007.00552.x 17850487

[B203] Savolainen M. H. Richie C. T. Harvey B. K. Männistö P. T. Maguire-Zeiss K. A. Myöhänen T. T. (2014). The beneficial effect of a prolyl oligopeptidase inhibitor, KYP-2047, on alpha-synuclein clearance and autophagy in A30P transgenic mouse. *Neurobiol. Dis.* 68 1–15. 10.1016/j.nbd.2014.04.003 24746855 PMC7254878

[B204] Sever S. Damke H. Schmid S. L. (2000). Dynamin:Gtp controls the formation of constricted coated pits, the rate limiting step in clathrin-mediated endocytosis. *J. Cell Biol.* 150 1137–1148. 10.1083/jcb.150.5.1137 10974001 PMC2175254

[B205] Shahmoradian S. H. Lewis A. J. Genoud C. Hench J. Moors T. E. Navarro P. P. (2019). Lewy pathology in Parkinson’s disease consists of crowded organelles and lipid membranes. *Nat. Neurosci.* 22 1099–1109. 10.1038/s41593-019-0423-2 31235907

[B206] Shaid S. Brandts C. H. Serve H. Dikic I. (2013). Ubiquitination and selective autophagy. *Cell Death Dif.* 20 21–30. 10.1038/cdd.2012.72 22722335 PMC3524631

[B207] Shayakhmetov D. M. Eberly A. M. Li Z. Y. Lieber A. (2005). Deletion of Penton RGD motifs affects the efficiency of both the internalization and the endosome escape of viral particles containing adenovirus serotype 5 or 35 Fiber Knobs. *J. Virol.* 79 1053–1061. 10.1128/jvi.79.2.1053-1061.2005 15613334 PMC538548

[B208] Shelke G. V. Yin Y. Jang S. C. Lässer C. Wennmalm S. Hoffmann H. J. (2019). Endosomal signalling via exosome surface TGFβ-1. *J. Extracell. Vesicles* 8:1650458. 10.1080/20013078.2019.1650458 31595182 PMC6764367

[B209] Shen W.-C. Li H.-Y. Chen G.-C. Chern Y. Tu P.-H. (2015). Mutations in the ubiquitin-binding domain of OPTN/optineurin interfere with autophagy-mediated degradation of misfolded proteins by a dominant-negative mechanism. *Autophagy* 11 685–700. 10.4161/auto.36098 25484089 PMC4502753

[B210] Shibata M. Lu T. Furuya T. Degterev A. Mizushima N. Yoshimori T. (2006). Regulation of intracellular accumulation of mutant huntingtin by beclin 1. *J. Biol. Chem.* 281 14474–14485. 10.1074/jbc.m600364200 16522639

[B211] Shim S. Y. Karri S. Law S. Schatzl H. M. Gilch S. (2016). Prion infection impairs lysosomal degradation capacity by interfering with rab7 membrane attachment in neuronal cells. *Sci. Rep.* 6:21658. 10.1038/srep21658 PMC4749993 26865414

[B212] Sikorska B. Liberski P. P. Giraud P. Kopp N. Brown P. (2004). Autophagy is a part of ultrastructural synaptic pathology in Creutzfeldt-Jakob disease: a brain biopsy study. *Int. J. Biochem. Cell Biol.* 36 2563–2573. 10.1016/j.biocel.2004.04.014 15325593

[B213] Singleton A. B. Farrer M. Johnson J. Singleton A. Hague S. Kachergus J. (2003). alpha-Synuclein locus triplication causes Parkinson’s disease. *Science* 302:841. 10.1126/science.1090278 14593171

[B214] Skog J. Wurdinger T. van Rijn S. Meijer D. H. Gainche L. Sena-Esteves M. (2008). Glioblastoma microvesicles transport RNA and proteins that promote tumour growth and provide diagnostic biomarkers. *Nat. Cell Biol.* 10 1470–1476. 10.1038/ncb1800 19011622 PMC3423894

[B215] Skotland T. Sagini K. Sandvig K. Llorente A. (2020). An emerging focus on lipids in extracellular vesicles. *Adv. Drug Deliv. Rev.* S0169-409X, 30014–30014. 10.1016/j.addr.2020.03.002 32151658

[B216] Song M. Wang Y. Shang Z.-F. Liu X.-D. Xie D.-F. Wang Q. (2016). Bystander autophagy mediated by radiation-induced exosomal miR-7-5p in non-targeted human bronchial epithelial cells. *Sci. Rep.* 6: 30165. 10.1038/srep30165 PMC4945935 27417393

[B217] Soto C. Pritzkow S. (2018). Protein misfolding, aggregation, and conformational strains in neurodegenerative diseases. *Nat. Neurosci.* 21 1332–1340. 10.1038/s41593-018-0235-9 30250260 PMC6432913

[B218] Spencer B. Potkar R. Trejo M. Rockenstein E. Patrick C. Gindi R. (2009). Beclin 1 Gene Transfer activates autophagy and ameliorates the neurodegenerative pathology in a-synuclein models of parkinson’s and lewy body diseases. *J. Neurosci.* 29 13578–13588. 10.1523/jneurosci.4390-09.2009 19864570 PMC2812014

[B219] Spilman P. Podlutskaya N. Hart M. J. Debnath J. Gorostiza O. Bredesen D. (2010). Inhibition of mTOR by rapamycin abolishes cognitive deficits and reduces Amyloid-β levels in a mouse model of alzheimer’s disease. *PLoS One* 5:e9979. 10.1371/journal.pone.0009979 20376313 PMC2848616

[B220] Srivastava K. R. Lapidus L. J. (2017). Prion protein dynamics before aggregation. *Proc. Natl. Acad. Sci. U. S. A.* 114 3572–3577. 10.1073/pnas.1620400114 28320943 PMC5389334

[B221] Staring J. Raaben M. Brummelkamp T. R. (2018). Viral escape from endosomes and host detection at a glance. *J. Cell Sci.* 131:jcs216259. 10.1242/jcs.216259 30076240

[B222] Steele J. W. Lachenmayer M. L. Ju S. Stock A. Liken J. Kim S. H. (2013). Latrepirdine improves cognition and arrests progression of neuropathology in an Alzheimer’s mouse model. *Mol. Psychiatry* 18 889–897. 10.1038/mp.2012.106 22850627 PMC3625697

[B223] Steenbeek S. C. Pham T. V. Ligt J. Zomer A. Knol J. C. Piersma S. R. (2018). Cancer cells copy migratory behavior and exchange signaling networks via extracellular vesicles. *EMBO J.* 37:e98357. 10.15252/embj.201798357 PMC6068466 29907695

[B224] Steinman R. M. (1983). Endocytosis and the recycling of plasma membrane. *J. Cell Biol.* 96 1–27. 6298247 10.1083/jcb.96.1.1 PMC2112240

[B225] Sterzenbach U. Putz U. Low L.-H. Silke J. Tan S.-S. Howitt J. (2017). Engineered exosomes as vehicles for biologically active proteins. *Mol. Ther.* 25 1269–1278. 10.1016/j.ymthe.2017.03.030 28412169 PMC5474961

[B226] Stolz A. (2014). Ernst, and I. Dikic, Cargo recognition and trafficking in selective autophagy. *Nat. Cell Biol.* 16 495–501. 10.1038/ncb2979 24875736

[B227] Stuart L. M. Ezekowitz R. A. B. (2005). Phagocytosis. *Immunity* 22 539–550. 15894272 10.1016/j.immuni.2005.05.002

[B228] Sullivan A. L. Grasso J. A. Weintraub L. R. (1976). Micropinocytosis of transferrin by developing red cells: an electron-microscopic study utilizing ferritin-conjugated transferrin and ferritin-conjugated antibodies to transferrin. *Blood* 47 133–143. 10.1182/blood.v47.1.133.bloodjournal471133 1244908

[B229] Svensson K. J. Christianson H. C. Wittrup A. Bourseau-Guilmain E. Lindqvist E. Svensson L. M. (2013). Exosome Uptake Depends on ERK1/2-heat shock protein 27 signaling and lipid raft-mediated endocytosis negatively regulated by Caveolin-1. *J. Biol. Chem.* 288 17713–17724. 10.1074/jbc.m112.445403 23653359 PMC3682571

[B230] Swanson J. A. (2008). Shaping cups into phagosomes and macropinosomes. *Nat. Rev. Mol. Cell Biol.* 9 639–649. 10.1038/nrm2447 18612320 PMC2851551

[B231] Swanson J. A. Watts C. (1995). Macropinocytosis. *Trends Cell Biol.* 5 424–428. 14732047 10.1016/s0962-8924(00)89101-1

[B232] Taelman V. F. Dobrowolski R. Plouhinec J. L. Fuentealba L. C. Vorwald P. P. Gumper I. (2010). Wnt signaling requires sequestration of glycogen synthase kinase 3 inside multivesicular endosomes. *Cell* 143 1136–1148. 10.1016/j.cell.2010.11.034 21183076 PMC3022472

[B233] Tanaka M. Machida Y. Niu S. Ikeda T. Jana N. R. Doi H. (2004). Trehalose alleviates polyglutamine-mediated pathology in a mouse model of Huntington disease. *Nat. Med.* 10 148–154. 10.1038/nm985 14730359

[B234] Tanida I. (2011). Autophagosome formation and molecular mechanism of autophagy. *Antioxid. Redox Signal.* 14 2201–2214. 10.1089/ars.2010.3482 20712405

[B235] Terry R. D. (1963). The fine sctructure of neurofibrillary tangles in Alzheimer’s disease. *J. Neuropathol. Exp. Neurol.* 22 629–642. 14069842 10.1097/00005072-196310000-00005

[B236] Théry C. Boussac M. Véron P. Ricciardi-Castagnoli P. Raposo G. Garin J. (2001). Proteomic analysis of dendritic cell-derived exosomes: a secreted subcellular compartment distinct from apoptotic vesicles. *J. Immunol.* 166 7309–7318. 10.4049/jimmunol.166.12.7309 11390481

[B237] Théry C. Regnault A. Garin J. Wolfers J. Zitvogel L. Ricciardi-Castagnoli P. (1999). Molecular characterization of dendritic cell-derived exosomes. *J. Cell Biol.* 147 599–610. 10.1083/jcb.147.3.599 10545503 PMC2151184

[B238] Thompson A. G. Gray E. Heman-Ackah S. M. Mäger I. Talbot K. Andaloussi S. E. (2016). Extracellular vesicles in neurodegenerative disease — pathogenesis to biomarkers. *Nat. Rev. Neurol.* 12 346–357. 10.1038/nrneurol.2016.68 27174238

[B239] Tian T. Zhu Y.-L. Zhou Y.-Y. Liang G.-F. Wang Y.-Y. Hu F.-H. (2014a). Exosome Uptake through Clathrin-mediated endocytosis and macropinocytosis and mediating miR-21 delivery. *J. Biol. Chem.* 289:22258–22267. 10.1074/jbc.m114.588046 24951588 PMC4139237

[B240] Tian Y. Li S. Song J. Ji T. Zhu M. Anderson G. J. (2014b). delivery platform using engineered natural membrane vesicle exosomes for targeted tumor therapy. *Biomaterials* 35 2383–2390. 10.1016/j.biomaterials.2013.11.083 24345736

[B241] Tooze S. A. Yoshimori T. (2010). The origin of the autophagosomal membrane. *Nat. Cell Biol.* 12 831–835. 10.1038/ncb0910-831 20811355

[B242] Tsujimura A. Taguchi K. Watanabe Y. Tatebe H. Tokuda T. Mizuno T. (2015). Lysosomal enzyme cathepsin B enhances the aggregate forming activity of exogenous α-synuclein fibrils. *Neurobiol. Dis.* 73 244–253. 10.1016/j.nbd.2014.10.011 25466281

[B243] Uversky V. N. (2007). Neuropathology, biochemistry, and biophysics of ?-synuclein aggregation. *J. Neurochem.* 130 17–37. 10.1111/j.1471-4159.2007.04764.x 17623039

[B244] Valadi H. Ekström K. Bossios A. Sjöstrand M. Lee J. J. Lötvall J. O. (2007). Exosome-mediated transfer of mRNAs and microRNAs is a novel mechanism of genetic exchange between cells. *Nat. Cell Biol.* 9 654–659. 10.1038/ncb1596 17486113

[B245] Valionyte E. Yang Y. Roberts S. L. Kelly J. Lu B. Luo S. (2020). Lowering mutant huntingtin levels and Toxicity: autophagy-endolysosome pathways in huntington’s disease. *J. Mol. Biol.* 432 2673–2691. 10.1016/j.jmb.2019.11.012 31786267

[B246] Van Dongen H. M. Masoumi N. Witwer K. W. Pegtel D. M. (2016). Extracellular vesicles exploit viral entry routes for cargo delivery. *Microbiol. Mol. Biol. Rev.* 80 369–386. 10.1128/mmbr.00063-15 26935137 PMC4867369

[B247] van Niel G. Charrin S. Simoes S. Romao M. Rochin L. Saftig P. (2011). The Tetraspanin CD63 Regulates ESCRT-independent and -dependent endosomal sorting during Melanogenesis. *Dev. Cell* 21 708–721. 10.1016/j.devcel.2011.08.019 21962903 PMC3199340

[B248] van Niel G. D’Angelo G. Raposo G. (2018). Shedding light on the cell biology of extracellular vesicles. *Nat. Rev. Mol. Cell Biol.* 19 213–228. 10.1038/nrm.2017.125 29339798

[B249] Vella L. Sharples R. Lawson V. Masters C. Cappai R. Hill A. (2007). Packaging of prions into exosomes is associated with a novel pathway of PrP processing. *J. Pathol.* 211 582–590. 10.1002/path.2145 17334982

[B250] Veltman D. M. Williams T. D. Bloomfield G. Chen B.-C. Betzig E. Insall R. H. (2016). A plasma membrane template for macropinocytic cups. *Elife* 5:e20085. 10.7554/eLife.20085 PMC5154761 27960076

[B251] Verweij F. J. Revenu C. Arras G. Dingli F. Loew D. Pegtel D. M. (2019). Live tracking of inter-organ communication by endogenous exosomes in vivo. *Dev. Cell* 48 e573–e589. 10.1016/j.devcel.2019.01.004 30745143

[B252] von Kleist L. Stahlschmidt W. Bulut H. Gromova K. Puchkov D. Robertson M. J. (2011). Role of the clathrin terminal domain in regulating coated pit dynamics revealed by small molecule inhibition. *Cell* 146 471–484. 10.1016/j.cell.2011.06.025 21816279

[B253] Wang B. Xing D. Zhu Y. Dong S. Zhao B. (2019). The state of exosomes research: a global visualized analysis. *BioMed Res. Int.* 2019:1495130. 10.1155/2019/1495130 PMC6470441 31073519

[B254] Wang L. H. Rothberg K. G. Anderson R. G. (1993). Mis-assembly of clathrin lattices on endosomes reveals a regulatory switch for coated pit formation. *J. Cell Biol.* 123 1107–1117. 10.1083/jcb.123.5.1107 8245121 PMC2119875

[B255] Wang Z. Tiruppathi C. Minshall R. D. Malik A. B. (2009). Size and dynamics of caveolae studied using nanoparticles in living endothelial cells. *ACS Nano* 3 4110–4116. 10.1021/nn9012274 19919048 PMC3643811

[B256] Watson L. S. Hamlett E. D. Stone T. D. Sims-Robinson C. (2019). Neuronally derived extracellular vesicles: an emerging tool for understanding Alzheimer’s disease. *Mol. Neurodegener.* 14:22. 10.1186/s13024-019-0317-5 PMC6558712 31182115

[B257] Wegmann S. Bennett R. E. Delorme L. Robbins A. B. Hu M. MacKenzie D. (2019). Experimental evidence for the age dependence of tau protein spread in the brain. *Sci. Adv.* 5:eaaw6404. 10.1126/sciadv.aaw6404 31249873 PMC6594764

[B258] Wesselborg S. Stork B. (2015). Autophagy signal transduction by ATG proteins: from hierarchies to networks. *Cell. Mol. Life Sci.* 72 4721–4757. 10.1007/s00018-015-2034-8 26390974 PMC4648967

[B259] Williams C. Pazos R. Royo F. González E. Roura-Ferrer M. Martinez A. (2019). Assessing the role of surface glycans of extracellular vesicles on cellular uptake. *Sci. Rep.* 9:11920. 10.1038/s41598-019-48499-1 PMC6695415 31417177

[B260] Williams C. Royo F. Aizpurua-Olaizola O. Pazos R. Boons G.-J. Reichardt N.-C. (2018). Glycosylation of extracellular vesicles: current knowledge, tools and clinical perspectives. *J. Extracell. Vesicles* 7:1442985. 10.1080/20013078.2018.1442985 29535851 PMC5844028

[B261] Willox A. K. Sahraoui Y. M. E. Royle S. J. (2014). Non-specificity of Pitstop 2 in clathrin-mediated endocytosis. *Biol. Open* 3 326–331. 10.1242/bio.20147955 24705016 PMC4021354

[B262] Winchester B. (2005). Lysosomal metabolism of glycoproteins. *Glycobiology* 15 1R–15R. 15647514 10.1093/glycob/cwi041

[B263] Wong E. Cuervo A. M. (2010). Autophagy gone awry in neurodegenerative diseases. *Nat. Neurosci.* 13 805–811. 10.1038/nn.2575 20581817 PMC4038747

[B264] Xie Z. Klionsky D. J. (2007). Autophagosome formation: core machinery and adaptations. *Nat. Cell Biol.* 9 1102–1109. 10.1038/ncb1007-1102 17909521

[B265] Xiong J. (2015). Atg7 in development and disease: panacea or Pandora’s Box? *Protein Cell* 6 722–734. 10.1007/s13238-015-0195-8 26404030 PMC4598325

[B266] Yamamoto A. Tagawa Y. Yoshimori T. Moriyama Y. Masaki R. Tashiro Y. (1998). Bafilomycin A1 prevents maturation of autophagic vacuoles by inhibiting fusion between autophagosomes and lysosomes in rat hepatoma cell line, H-4-II-E cells. *Cell Struct. Funct.* 23 33–42. 10.1247/csf.23.33 9639028

[B267] Yang D.-S. Stavrides P. Mohan P. S. Kaushik S. Kumar A. Ohno M. (2011). Reversal of autophagy dysfunction in the TgCRND8 mouse model of Alzheimer’s disease ameliorates amyloid pathologies and memory deficits. *Brain* 134 258–277. 10.1093/brain/awq341 21186265 PMC3009842

[B268] Yang S.-T. Kreutzberger A. J. B. Lee J. Kiessling V. Tamm L. K. (2016). The role of cholesterol in membrane fusion. *Chem. Phys. Lipids* 199 136–143. 10.1016/j.chemphyslip.2016.05.003 27179407 PMC4972649

[B269] Yang Y. Hong Y. Cho E. Kim G. B. Kim I.-S. (2018). Extracellular vesicles as a platform for membrane-associated therapeutic protein delivery. *J. Extracell. Vesicles* 7:1440131. 10.1080/20013078.2018.1440131 29535849 PMC5844050

[B270] Yao Z. Qiao Y. Li X. Chen J. Ding J. Bai L. (2018). Exosomes exploit the virus entry machinery and pathway to transmit alpha interferon-induced antiviral activity. *J. Virol.* 92 e1578–e1518. 10.1128/JVI.01578-18 PMC6258946 30282711

[B271] Yarmola E. G. Somasundaram T. Boring T. A. Spector I. Bubb M. R. (2000). Actin-latrunculin A structure and function:differential modulation of actin-binding protein function by latrunculin A. *J. Biol. Chem.* 275 28120–28127. 10859320 10.1074/jbc.M004253200

[B272] Yoon Y.-S. Cho E.-D. Ahn W. J. Lee K. W. Lee S.-J. Lee H.-J. (2017). Is trehalose an autophagic inducer? Unraveling theroles of non-reducing disaccharides on autophagic fluxand alpha-synuclein aggregation. *Cell Death Dis.* 8:e3091. 10.1038/cddis.2017.501 28981090 PMC5682667

[B273] Zhang Z. Song M. Liu X. Kang S. S. Kwon I.-S. Duong D. M. (2014). Cleavage of tau by asparagine endopeptidase mediates the neurofibrillary pathology in Alzheimer’s disease. *Nat. Med.* 20 1254–1262. 25326800 10.1038/nm.3700 PMC4224595

[B274] Zhou Z. Xue Q. Wan Y. Yang Y. Wang J. Hung T. (2011). Lysosome-associated membrane glycoprotein 3 is involved in influenza A virus replication in human lung epithelial (A549) cells. *Virol. J.* 8:384. 10.1186/1743-422x-8-384 21810281 PMC3162545

[B275] Zientara-Rytter K. Subramani S. (2019). The roles of ubiquitin-binding protein shuttles in the degradative fate of ubiquitinated proteins in the ubiquitin-proteasome system and autophagy. *Cells* 8:40. 10.3390/cells8010040 30634694 PMC6357184

[B276] Zomer A. Maynard C. Frederik J. V. Kamermans A. Schäfer R. Beerling E. (2015). In Vivo imaging reveals extracellular vesicle-mediated phenocopying of metastatic behavior. *Cell* 161 1046–1057. 10.1016/j.cell.2015.04.042 26000481 PMC4448148

